# Modelling normal age-related changes in individual retinal layers using location-specific OCT analysis

**DOI:** 10.1038/s41598-020-79424-6

**Published:** 2021-01-12

**Authors:** Matt Trinh, Vincent Khou, Barbara Zangerl, Michael Kalloniatis, Lisa Nivison-Smith

**Affiliations:** 1 Centre for Eye Health, University of New South Wales, Sydney, 2052 Australia; 2 School of Optometry and Vision Science, University of New South Wales, Sydney, 2052 Australia

**Keywords:** Translational research, Cells

## Abstract

Current descriptions of retinal thickness across normal age cohorts are mostly limited to global analyses, thus overlooking spatial variation across the retina and limiting spatial analyses of retinal and optic nerve disease. This retrospective cross-sectional study uses location-specific cluster analysis of 8 × 8 macular average grid-wise thicknesses to quantify topographical patterns and rates of normal, age-related changes in all individual retinal layers of 253 eyes of 253 participants across various age cohorts (n = 23–69 eyes per decade). Most retinal layers had concentric spatial cluster patterns except the retinal nerve fibre layer (RNFL) which displayed a nasal, asymmetric radial pattern. Age-related thickness decline mostly occurred after the late 4th decade, described by quadratic regression models. The ganglion cell layer (GCL), inner plexiform layer (IPL), inner nuclear layer (INL), and outer nuclear layer + Henle’s fibre layer (ONL_+HFL_) were significantly associated with age (*p* < 0.0001 to < 0.05), demonstrating similar rates of thickness decline (mean pooled slope =  − 0.07 µm/year), while the IS/OS had lesser mean pooled thickness slopes for all clusters (− 0.04 µm/year). The RNFL, OPL, and RPE exhibited no significant age-related thickness change, and the RNFL were significantly associated with sex. Analysis using spatial clusters compared to the ETDRS sectors revealed more extensive spatial definition and less variability in the former method. These spatially defined, clustered normative data and age-correction functions provide an accessible method of retinal thickness analysis with more spatial detail and less variability than the ETDRS sectors, potentially aiding the diagnosis and monitoring of retinal and optic nerve disease.

## Introduction

Normal ageing sees the loss of retinal neurons such as photoreceptors, bipolar cells, and ganglion cells^1–4^. As such, a persistent issue in retinal disease biology is accurately differentiating retinal thickness changes due to disease versus the normal ageing process. Several studies have attempted to develop normative age-related databases for individual retinal layers using OCT^5–14^, however, the majority of these databases are limited by a combination of several factors. Firstly, the small sample sizes in some studies mean that they do not adequately account for demographic characteristics such as sex and age, which can lead to significant variability in OCT thickness measurements^15^. Secondly, if spatial variation in retinal layer thickness is considered at all, spatial localisation is then defined over arbitrary spatial maps such as the Early Treatment Diabetic Retinopathy Study (ETDRS) sectors which assume a symmetric, concentric topography of ageing changes. Finally, linear regression models have been applied in an assumptive manner to data^5–13^ which has otherwise shown non-linear decline in other studies, e.g. in the GCL and rod bipolar cells of the INL^2,3,16^. These assumptions may then contribute to the variable rates of change that have been reported, including conflicting trends of thickening and thinning that have emerged in the photoreceptor layer^10–14,17^.

To overcome the discussed limitations, our research group has recently developed a method of OCT analysis with high-density sampling as compared to the ETDRS sectors, applied to a large cohort (to mitigate inter-individual variability) to quantify the topographical patterns and rates of normal, age-related thickness changes. Specifically, we have shown that in the macula GCL, the topography and subsequent non-linear age-correction functions for normal eyes’ retinal layer thicknesses can be derived from the ‘clustering’ of locations within the macula that demonstrate statistically similar thicknesses and age-related changes^16,18^. Spatial clustering provides a less arbitrary definition of retinal topography due to its post-hoc analysis. The subsequent non-linear regression models derived from these clusters have demonstrated statistical superiority over linear models^16,18^ and also more accurately reflect physiological decline in retinal ageing^2,3,16^. Comparisons between clustered macula structure–function models have also demonstrated superior prediction over traditional point-wise macula structure–function models^16^. This methodology has been further verified against diseased eyes, in applications such as being able to differentiate RNFL thickness changes between severity stages of normal tension glaucoma eyes^19^, the automated analysis of pathological intra-retinal cavities^20^, differentiating GCL thickness changes in AMD versus normal eyes^21^, and the detection of greater visual field defects in AMD eyes using cluster analysis as opposed to point-wise analysis^22^.

Beyond the RNFL and GCL^16,23,24^, spatial clustering has not been applied to other retinal layers. Development of an OCT thickness normative database that includes cluster-based spatial localisation for all retinal layers would provide an accessible method of intra- and inter-retinal layer comparisons in vivo, as well as enable future development of clustered macula structure–function models in order to aid the diagnosis and monitoring of a number of retinal and optic nerve diseases. This database would also enable the development of age-correction functions, which have been previously applied to GCL thickness data of diseased and normal eyes, demonstrating that age-corrected analyses were on par with age-matched analyses^21^. Further validation in all retinal layers may then be useful in future studies where cohorts are heterogenous or under-sampled, or in clinical practice where the expected output normative age may be calculated from the input retinal layer thickness.

Thus, in this retrospective cross-sectional study, we apply the high-density sampling method of OCT macular data to a large cohort of normal eyes for each individual retinal layer. From this, we quantify the topographical patterns and rates of normal, age-related thickness changes in each retinal layer and corroborate previous retinal clustering methods^16,23,24^. We also aim to develop age-correction functions for each retinal layer’s clustered data, providing an accessible tool to eventually compare to diseased eyes.

## Methods

### Study population

A total of 253 participants across various age cohorts were included in the study, previously identified by Tong et al.^16^ All participants and their data were obtained through retrospective analysis of records from 05/25/2012 to 02/28/2017 of patients attending the Centre for Eye Health (CFEH) Sydney, Australia. CFEH is a referral-only clinic providing advanced diagnostic eye testing and disease management by specially trained optometrists and ophthalmologists^25^. All patients had given prior written informed consent to use their de-identified data for research in accordance with the Declaration of Helsinki and approved by the Biomedical Human Research Ethics Advisory Panel of the University of New South Wales.

Complete characteristics of the cohort are described in Tong et al.^16^ Briefly, all participants met inclusion criteria, defined as: visual acuity better than 20/25 (logMAR < 0.1) for participants under 60 years old, or 20/32 (logMAR < 0.2) for participants over 60 years old, intraocular pressure < 22 mmHg in both eyes, spherical equivalent refractive error between + 3.00 and − 6.00 dioptres and astigmatism < 3.00 dioptres^26^, and no evidence of ocular disease including but not limited to glaucoma, diabetic retinopathy, AMD, or signs of significant sub-retinal or intra-retinal deposits, fluid, pigment, or vascular changes at the macula^27^.

### Image acquisition and data extraction

**Figure 1:**
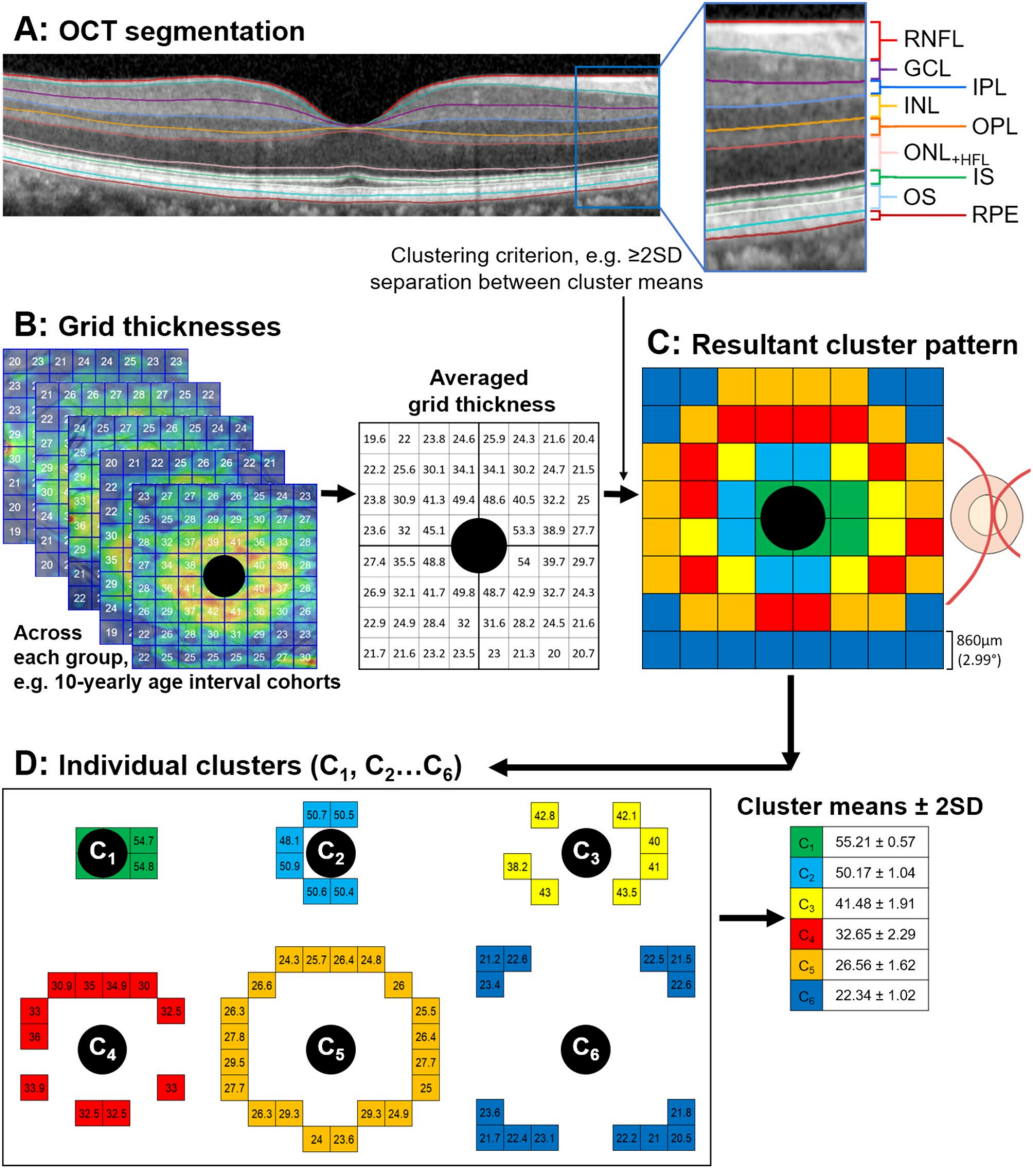
(**A**) Retinal layers segmented within the HRA/Spectralis Viewing module – manual correction was performed when necessary. (**B**) Individual retinal layer thicknesses across each group (i.e. variables that were significantly associated with average retinal layer thickness, such as age or sex). The Spectralis 8 × 8 grids are centred on the fovea with each grid location covering 0.74 mm^2^ (approximately 3° × 3°). Values presented are a sample of GCL thicknesses in µm. For each group, averaged thickness were then determined at each grid location (*middle*). (**C**) Values were then clustered by statistically similar thicknesses based on applied criterion of at least 2SD separation (d′ ≥ 2) between cluster means. The resultant spatial cluster topography is generated by the various pseudo-colours. Note all images are in right eye format, demonstrated by the location of the optic nerve. Scale bar is provided in the bottom left. (**D**) A visual and numerical break-down of the individual clusters, demonstrating a concentric pattern. GCL, ganglion cell layer; IPL, inner plexiform layer; INL, inner nuclear layer; OPL, outer plexiform layer; ONL_+HFL_, outer nuclear layer + Henle’s fibre layer; IS, photoreceptor inner segment layer; OS, photoreceptor outer segment layer; RPE, retinal pigment epithelium. C_1…6_ = Clusters 1…6.

OCT macular cube scans (61 B-scans covering an area of 8600 µm × 7167 µm or 30° × 25°) were acquired with Spectralis SD-OCT (Heidelberg Engineering, Heidelberg, Germany) as previously described^16,18^. The preset scan pattern were selected for commercial accessibility and includes the second highest number of retinal B-scans available as a preset option on the Spectralis SD-OCT, with the same or denser spacing (120 µm) as compared to other normative databases using the Spectralis SD-OCT^28–32^. Note, the preset scan pattern with the highest number of B-scans produces a much lower automatic real time mean function and significantly lower image quality, and hence was not used. The difference in horizontal versus vertical B-scan resolution has been shown not to have a significant impact on thickness outcomes^33^. Scans with significant artefacts or signal strength of lower than 15 dB were excluded. If multiple scans were available for each participant, the earliest scan meeting image quality criteria was selected. Scans were corrected for ocular tilt, and automatically segmented into each individual retinal layer using the HRA/Spectralis Viewing Module 6.9.5.0 (Heidelberg Engineering, Heidelberg, Germany; www.HeidelbergEngineering.com). The RNFL, IPL, INL, OPL, ONL_+HFL_, IS/OS, and RPE were reviewed (Fig. 1A) in all scans and corrected where necessary. Data regarding the GCL were obtained from Tong et al. and included in this study for completeness^16^. Manual correction of the OCT segmentation were performed in approximately 50% of all B-scans and adjustments were mostly minor corrections of segmentation lines that had erroneously shifted due to overlying blood vessel shadowing or ambiguity at Henle’s fibre layer. Agreement between authors MT and VK regarding manual correction were selected as the ‘ground-truth’ in concordance with other studies that have used manual correction as the benchmark against automated OCT segmentation protocol^34^. No B-scan segmentation were unresolvable between MT and VK.

The ELM and IS/OS segmentation lines were adjusted to fit into the middle, rather than the inner or outer borders. For scans where manual correction was not possible, grid locations were excluded from the analysis. If an eye had more than 10% of grid locations missing, the eye was excluded from the analysis^18^. The total of 253 participants that were included had satisfied this criterion, as previously identified by Tong et al.^16^.

Individual retinal layer thicknesses were then extracted from across the macula cube area (8600 µm × 7167 µm or 30° × 25°) as 64 measurements within an 8 × 8 grid (6880 µm × 6880 µm or 24° × 24°) centred on the fovea (each grid location covering 0.74 mm^2^ [approximately 860 µm × 860 µm or 3° × 3°]; Fig. 1B). 8 × 8 grids were selected for commercial availability, facilitating future comparisons against our normative data without the need for customised extraction tools. The central 4 grids of the GCL, IPL, INL, and OPL systematically underestimate true foveal thicknesses due to cellular displacement secondary to Henle’s fibres. As such, GCL thickness measurements were averaged across an additional 4 locations within a 1-degree surrounding area as described by Tong et al. and Yoshioka et al.^16,18^ Similar adjustments for the IPL, INL, and OPL were unreliable and not yet validated^11,13^, as previous studies that have included this area demonstrate retinal thickness values that are disparate to all other intra-retinal-layer areas^10,12,14^. To ensure that this systematic under-estimation did not produce unreliable normative data, we chose to exclude the foveal region of the IPL, INL, and OPL^11,13^. Thus, the total number of grids analysed for each eye was 64 in the RNFL, GCL, ONL_+HFL_, IS/OS, and RPE, and 60 in the IPL, INL, and OPL.

### Definitions of the retinal layers

All retinal layers were segmented according to typical reflectance profiles as seen in other OCT studies^10–14^. While maintaining anatomical accuracy is ideal, there are some important discrepancies that accompany the use of OCT regarding the anatomical segmentation of retinal layers compared to histological studies:

- In the RNFL, the major retinal arterioles and venules are routinely included as part of the layer’s segmentation, and thus true cellular thickness may be slightly misrepresented.
- In the OPL and ONL, inconsistent reflectivity of Henle’s fibre layer (HFL) on OCT may lead to ambiguities in segmentation. Commonly, HFL is segmented as part of the ONL—criteria which we have also adopted in this study for consistency (Fig. 1A, ‘ONL_+HFL_’)—despite technically being part of the OPL.

### Spatial clustering

Multi-variable linear regression analysis were performed to determine which demographic variable(s), i.e. age, sex, ethnicity, spherical equivalent refraction, and/or best corrected visual acuity (BCVA), were significantly associated with average retinal thickness for each retinal layer. Grid locations for each individual retinal layer were then grouped by significant variables into spatial clusters of statistically similar thicknesses. For example: if age were considered to be a significant variable with regards to average GCL thickness, then grid-wise GCL thickness were clustered across 5- or 10-yearly age interval cohorts (Fig. 1B,C); similarly, if sex were considered to be a significant variable, then grid-wise thickness were clustered across female and male cohorts.

Previously, spatial clusters have been established to be robust for GCL and perimetric analysis regarding normal^16,18^ and diseased eyes^22,35,36^. In this study, hierarchical clustering was initially performed using within-groups linkage and squared Euclidean distance via SPSS Statistics Version 25.0 (IBM Corporation, Armonk, NY, USA), to determine the maximum number of clusters for each retinal layer. This number was then applied to k-means clustering for each retinal layer. For each resultant pattern in this study, statistically significant separability of clusters was verified by d′ = $\frac{{|x}_{1}-{x}_{2}|}{\sqrt{0.5\times \left({\sigma }_{1}^{ 2}+{\sigma }_{2}^{ 2}\right)}}$, whereby any cluster with d′ < 2 (indicating separation of cluster means by less than two-standard deviation (SD)) were merged until all cluster patterns were separated by d′ ≥ 2 (Fig. 1C,D)^36^. To relate our findings to a more commonly used retinal spatial template, we also compared our grid-wise clusters to the ETDRS sectors.

### Age regression analysis

Regression analysis comparing cluster thicknesses to age (represented by 5- or 10-yearly age interval cohorts) were then performed to explore location-specific age-related thickness changes for each retinal layer. For layers where age was not significantly associated with average retinal thickness, these analyses were not performed. Linear and quadratic functions were processed using GraphPad Prism Version 8.0 (San Diego, CA, USA). Bi-linear functions have also been considered previously, however, the abrupt decline following a ‘critical point’ was counterintuitive when considering the gradual nature of ageing in ocular processes^37,38^. Hence, quadratic functions were preferentially selected over bi-linear models, due to the former’s gradual approach towards the critical point which seemed more physiologically appropriate^16^. In cases where there were no significant differences between quadratic and linear fit, a quadratic fit was applied to maintain consistency with previous studies^16^, and to more accurately represent previous histological data regarding retinal cellular density changes with age^2,3,16^. Quadratic functions that infinitely approached linearity were considered to be linear. Vertex points for quadratic regression functions were derived from the equation *x* = − $\frac{b}{2a}$.

### Selection of final cluster patterns

Spatial clustering for each retinal layer resulted in multiple cluster patterns due to the two methods applied (hierarchical and k-means). Selection of the final cluster pattern for each retinal layer were based on the optimal age-related regression fit, i.e. the highest mean R^2^, lowest mean sum-of-squares, and/or lowest SD-of-residuals, or the lowest coefficient of variation in cases where age-related regression analysis did not apply. If comparisons were tied in statistical significance or resulted in no significant differences of *p* < 0.05, the more optimal fit or lower coefficient of variation were selected.

### Statistical analysis

Statistical analyses were performed using GraphPad Prism Version 8.0 and SPSS Statistics Version 25, with significance considered as *p* < 0.05. Multi-variable linear regression analysis were performed with backward step-wise elimination (i.e. removing non-significant variables from the regression model in a step-wise manner)^39^. Categorical variables were encoded as dichotomous values, e.g. females set as 1, males set as 0; Asian ethnicity set as 1, White ethnicity set as 0, where necessary. Multiple inter-group comparisons between clusters, e.g. females versus males, were performed using Mann Whitney *U*-tests with Bonferroni adjustment. For age regression analysis, comparisons within cluster patterns were performed using Kruskal–Wallis test with post-hoc Dunn’s multiple comparisons tests. Comparison of slopes within cluster patterns were performed using the equivalent of an ANCOVA test, and between cluster patterns using Brown-Forsythe test with post-hoc Dunnett’s multiple comparisons tests. Comparison of slopes to a hypothetical zero slope were performed using an F-test.

## Results

### Subject demographics

Two-hundred-and-fifty-three healthy, normal eyes from 253 participants were included in this study, with participant demographics provided in Table 1. Multi-variable linear regression accounting for age, sex, ethnicity, spherical equivalent refraction, and BCVA showed that age were significantly associated with average retinal thickness in the GCL (*p* < 0.01), IPL (*p* < 0.01), INL (*p* < 0.0001), ONL (*p* < 0.01), and IS/OS (*p* < 0.001), but not the RNFL, OPL, nor RPE (Table 2). There were no other co-variables significantly associated with average retinal thicknesses except for sex with regards to average RNFL thickness (*p* < 0.01).

**Table 1:** Participant demographics based on 10-yearly age interval cohorts.

Cohort	n	Age ± SD (range)	Sex M:F	Ethnicity	Spherical equivalent Rx ± SD	BCVA logMAR ± SD
All participants	253	50.26 ± 14.4 (20.21–84.91)	108:145	154 W, 93A, 5O	− 0.48 ± 1.73	0.01 ± 0.1
20–29	29	25.5 ± 2.99	11:18	13 W, 16A	− 1.45 ± 1.71	− 0.04 ± 0.06
30–39	26	34.15 ± 2.97	9:17	9 W, 17A	− 1.42 ± 1.72	− 0.02 ± 0.1
40–49	69	45.84 ± 2.77	25:44	39 W, 28A, 2O	− 0.73 ± 1.54	− 0.01 ± 0.08
50–59	66	54.8 ± 2.84	30:36	42 W, 20A, 3O	− 0.16 ± 1.43	0 ± 0.07
60–69	40	64.02 ± 2.89	19:21	32 W, 8A	− 0.03 ± 2.07	0.04 ± 0.1
70+	23	75.95 ± 4.43	14:9	19 W, 4A	0.83 ± 1.2	0.1 ± 0.1

Values are expressed as mean ± standard deviation (SD). Description of demographic characteristics of the study population for 5-yearly age intervals were previously reported by Tong et al.^16^.

W, White; A, Asian; O, Other; Rx, refraction; BCVA, best corrected visual acuity.

**Table 2:** Multi-variable regression characteristics of participant demographics versus average retinal thicknesses.

	Age (β)	*p*-value	Sex (β)	*p*-value
RNFL	–	–	1.82 (0.59, 3.05)	< 0.01
GCL	− 0.05 (− 0.07, − 0.03)	< 0.0001	–	–
IPL	− 0.02 (− 0.04, 0)	< 0.05	–	–
INL	− 0.04 (− 0.06, − 0.02)	< 0.0001	–	–
OPL	–	–	–	–
ONL_+HFL_	− 0.06 (− 0.1, − 0.01)	< 0.01	–	–
IS/OS	− 0.02 (− 0.04, − 0.01)	< 0.001	–	–
RPE	–	–	–	–

The coefficients (95% CI) *below* represent final significant values included in each multi-variable model for each layer, based on backward step-wise elimination of co-variables.

### Spatial topography and age regression analysis

Grid-wise thickness data for each retinal layer were grouped by significant variable into spatial clusters of statistically similar thickness profiles. Specifically from the multi-variable linear regression analysis above: the RNFL were grouped by sex; the GCL, IPL, INL, ONL, and IS/OS were grouped by age (represented by 5- or 10-yearly age interval cohorts); and the OPL and RPE were singularly grouped using the whole cohort. Two different clustering methods were applied (hierarchical and k-means), with resultant cluster patterns and their characteristics for each retinal layer seen in (Supplementary Tables 1–8).Overall, clustering for each retinal layer produced similar topographical patterns with a similar number of clusters, and in some cases identical patterns were noted. Comparison of goodness-of-fit or coefficient of variation characteristics were then used to select the final cluster pattern for each retinal layer. In all comparisons of 5- and 10-yearly age interval cohort data, the latter showed better regression fit. Both clustering methods (hierarchical and k-means) were viable for each retinal layer, and there was no consistent superior method with regards to optimal goodness-of-fit or coefficient of variation.

In applying quadratic functions to age-related regression analysis, this implied an initial increase in thickness before the vertex age, whereby a decline in thickness followed. The mean increase in thickness for all clusters fit with a quadratic function across all retinal layers was 0.39 µm equivalent to an annual increase of 0.03 µm/year, suggesting minimal marked increase before the vertex age. Further analysis of all slopes before the vertex age revealed no statistically significant differences from a zero slope (F-test, *p* > 0.07 to 0.98 for all comparisons).

### RNFL and GCL

**Figure 2:**
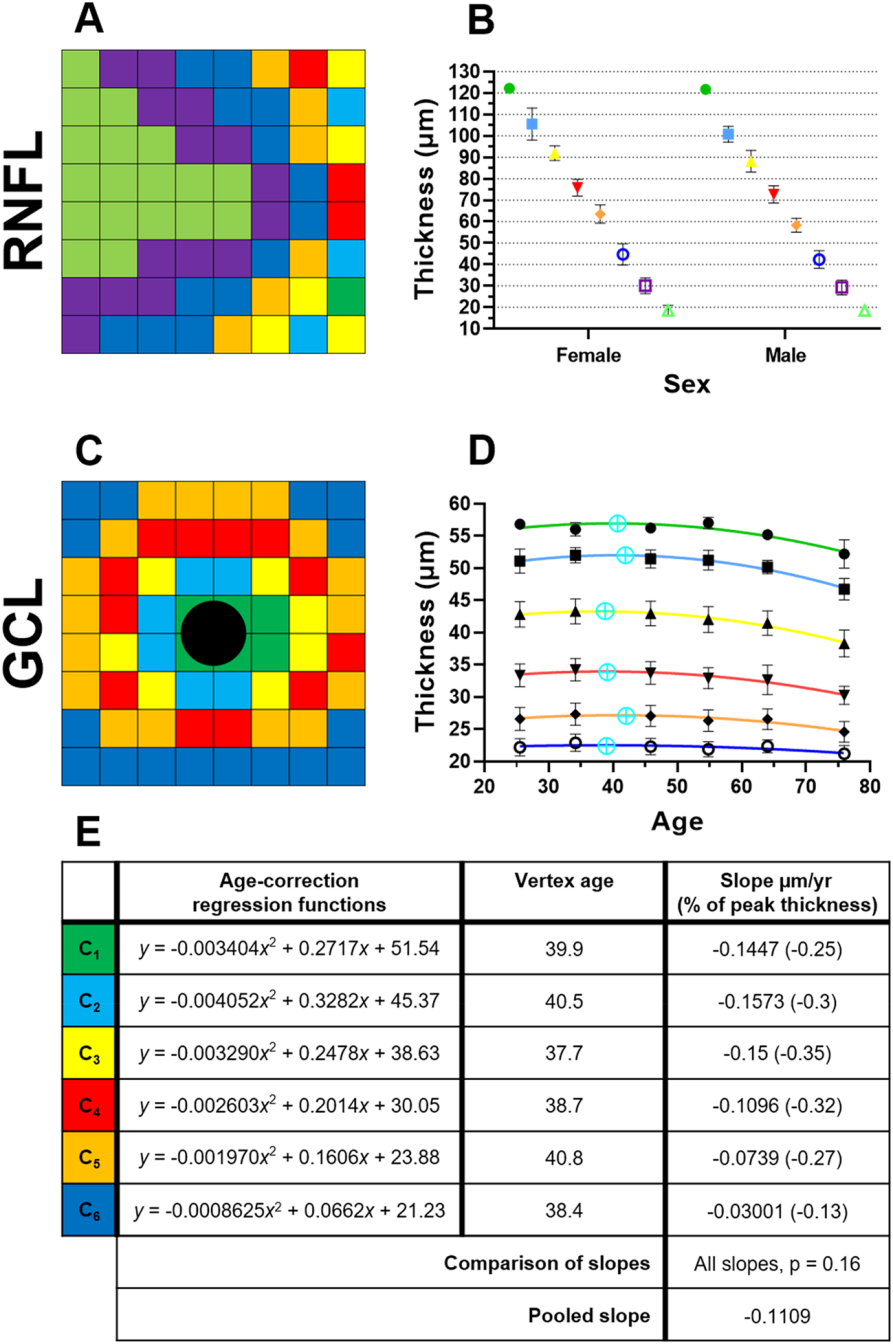
Spatial topography and age-related regression analysis where relevant in the (**A**,**B**) retinal nerve fibre layer (RNFL) and (**C–E**) ganglion cell layer (GCL), using location-specific cluster analysis. For each layer, the (**A,C**) spatial cluster patterns (in right eye format), (**B**,**D**) corresponding plots of cluster thickness versus groups, i.e. sex or age interval cohorts (cyan crosses denote vertex age), and (**E**) corresponding age-correction regression functions, vertex ages (if within the limits of this study’s cohort), and slopes as derived after the vertex age are presented. All slopes were significantly different from zero (*p* < 0.05). Central black circle in the GCL represents the excluded central four grids containing the fovea. Regression characteristics are in the forms *y* = a*x*^2^ + b*x* + c (quadratic) or *y* = m*x* + c (linear), where *x* = age, *y* = thickness (µm). ‘a’, ‘b’, and ‘m’ are co-efficients, and ‘c’ is a constant. Peak thicknesses were based off the vertex age for quadratic models, or *y*-intercept for linear models or cases where the vertex age was beyond the age limits of this study. In cases where post-vertex age included less than three averaged data points, the nearest neighbouring data points up to three averaged totals were included to minimise variability. Mean thickness ± SD of all plotted values are provided in (Supplementary Table 9).

Cluster analysis of the RNFL led to macula grid locations being assigned into eight different clusters that formed an asymmetric pattern, decreasing in thickness profile outwards from the optic nerve head at the nasal retina. Greater superior and inferior thicknesses were also observed (more so in the inferior clusters) compared to temporal thicknesses (Fig. 2A). As the RNFL were not significantly associated with age, this suggested no age-related change in RNFL thickness. However, as the RNFL were significantly associated with sex, clusters were instead analysed by sex. All clusters (C_1_–C_7_) except for C_8_ showed greater RNFL thicknesss in females compared to males (Fig. 2B), however these differences were not statistically significant (Mann Whitney *U*-tests; adjusted *p* = 0.04 to 0.97).

Analysis of the GCL led to six different clusters which formed a concentric pattern, with a slightly higher thickness profile superiorly (C_4_, C_5_) and nasally (C_1_, C_3_, C_4_) compared to inferiorly (C_4_–C_6_) and temporally (C_2_–C_5_), respectively (Fig. 2C). All clusters C_1_–C_6_ were fitted with downward quadratic functions, with decline in GCL thickness beginning between 37.7 to 40.8 years of age (Fig. 2D,E). The rate of age-related GCL thickness loss was not significantly different between all clusters (*p* = 0.16), thus the pooled thickness loss was calculated as − 0.11 µm/year.

### IPL, INL, and OPL

**Figure 3:**
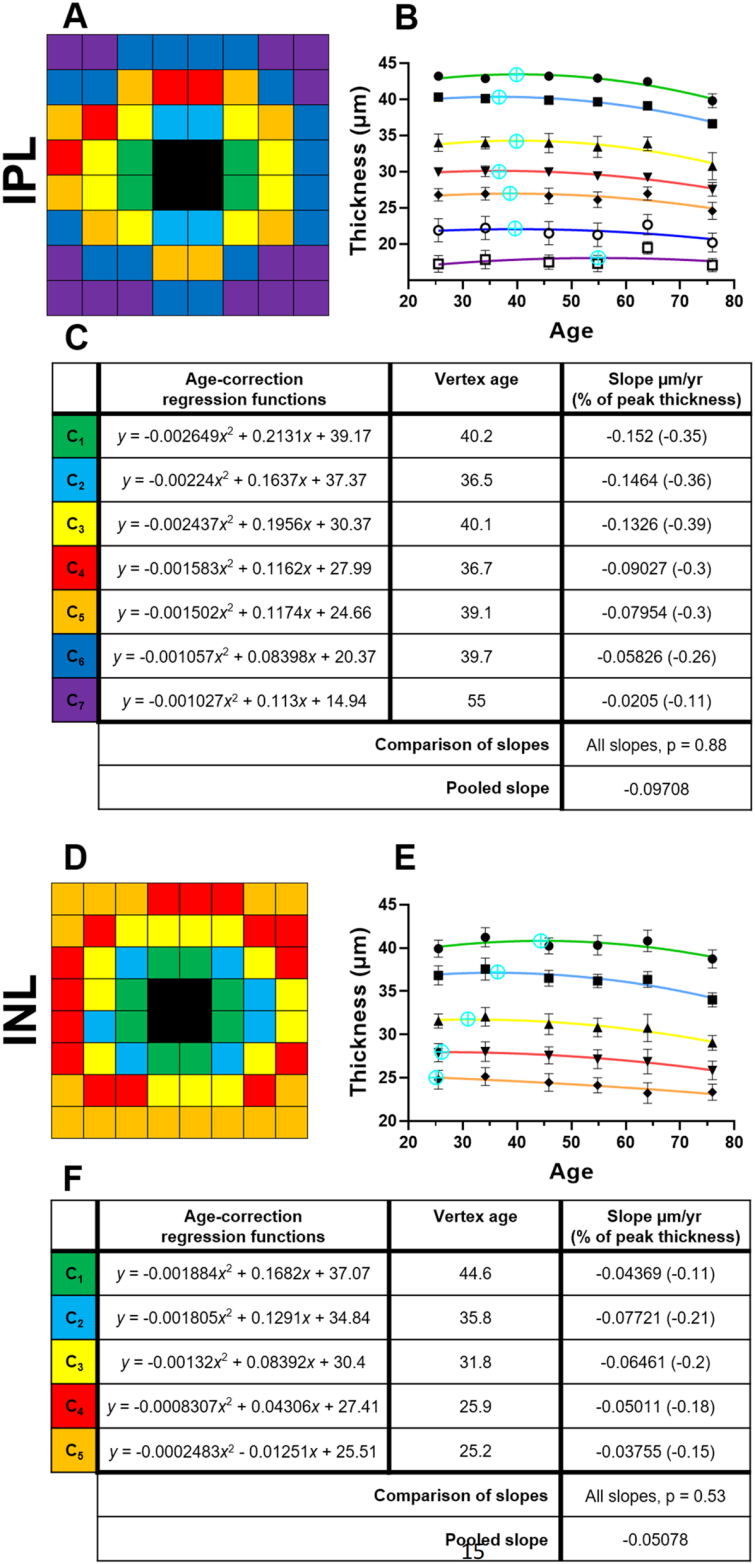

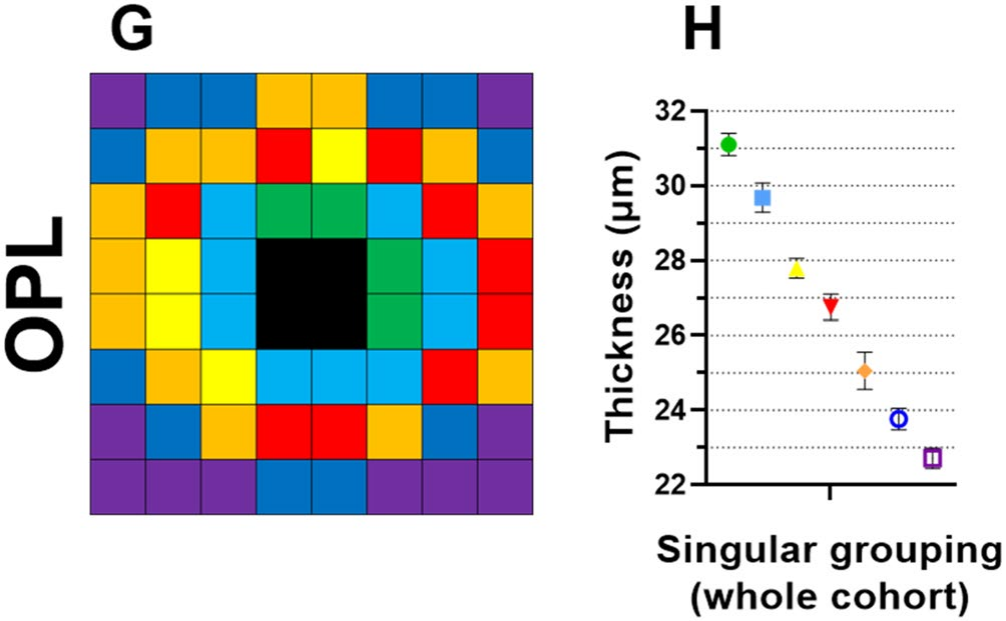
Spatial topography and age-related regression analysis where relevant in the (**A**–**C**) inner plexiform layer (IPL), (**D**–**F**) inner nuclear layer (INL), and (**G**,**H**) outer plexiform layer (OPL), using location-specific cluster analysis. For each layer, the (**A**,**D**,**G**) spatial cluster patterns, (**B**,**E**,**H**) corresponding plots of cluster thickness versus groups, i.e. age interval cohorts or singular grouping using the whole cohort, and (**C**,**F**) corresponding age-correction regression functions, vertex ages and slopes as derived after the vertex age are presented. All slopes were significantly different from zero (*p* < 0.05). Presentation as in Fig. 2.

For the IPL, INL, and OPL, all clustering patterns followed a relatively concentric arrangement, similar to the GCL (Fig. 3A,D,G). In the IPL, seven different clusters were identified with a slightly higher thickness profile superiorly (C_4_–C_6_) compared to inferiorly (C_5_–C_7_) (Fig. 3A). All IPL clusters were fitted with downward quadratic functions, with estimated IPL thickness loss beginning between 36.5 and 55 years of age (Fig. 3B,C) at a pooled loss of − 0.1 µm/year (based on no significant difference between all clusters, *p* = 0.88).

In the INL, five different clusters were identified with a slightly horizontal elongation of cluster topography (Fig. 3D). All clusters C_1_–C_5_ were fitted with downward quadratic functions, with decline beginning between 25.2 and 44.6 years of age (Fig. 3E,F). The rate of INL age-related thickness loss was not significantly different between all clusters (*p* = 0.53), and the pooled loss was calculated as − 0.05 µm/year.

Finally, for the OPL, clustering analyses identified seven statistically separable clusters, with a slightly higher thickness profile superiorly (C_1_–C_3_) and nasally (C_1_ and C_2_) versus inferiorly (C_2_–C_4_) and temporally (C_2_ and C_3_), respectively (Fig. 3G,H). As the OPL were not significantly associated with any variables including age, this suggested no age-related change in OPL thickness. This was confirmed in age regression analysis of all OPL cluster thicknesses (data not shown).

### ONL_+HFL_, IS/OS, and RPE

**Figure 4:**
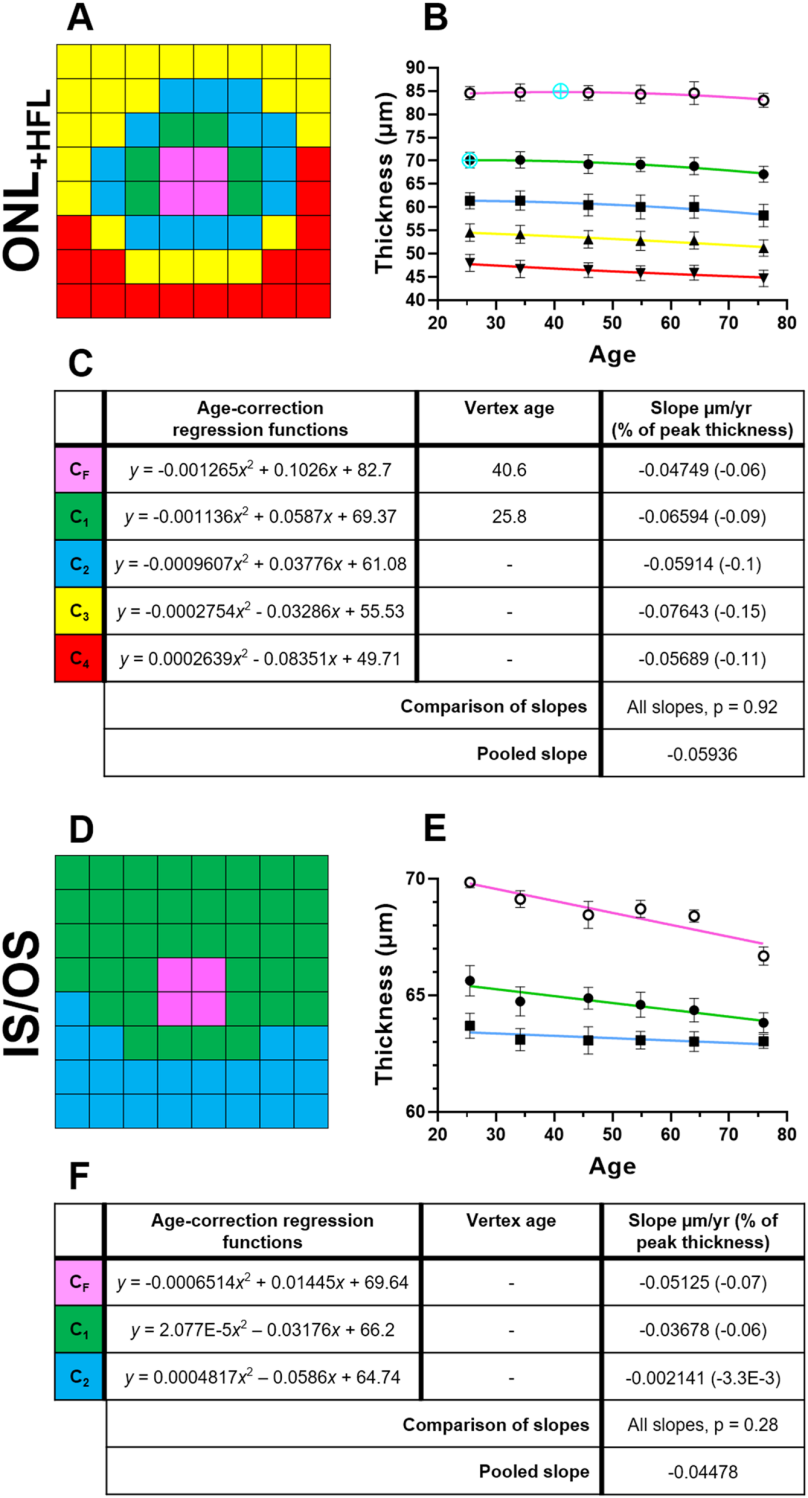

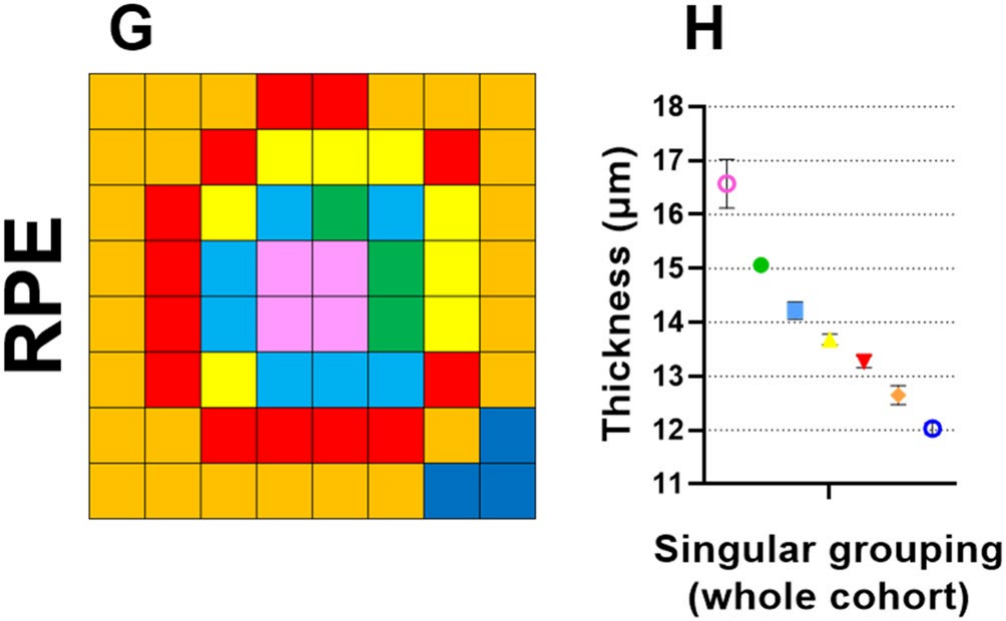
Spatial topography and age-related regression analysis where relevant in the (**A**–**C**) outer nuclear layer with Henle’s fibre layer (ONL_+HFL_), (**D**–**F**) photoreceptor inner-segment and outer-segment layer (IS/OS), and (**G**–**I**) retinal pigment epithelium (RPE), using location-specific cluster analysis. For each layer, the (**A**,**D**,**G**) spatial cluster patterns (in right eye format), (**B**,**E**,**H**) corresponding plots of cluster thickness versus groups, i.e. age interval cohorts or singular grouping using the whole cohort, and (**C**,**F**) corresponding age-correction regression functions, vertex ages), and slopes as derived after the vertex age are presented. All slopes were significantly different from zero (*p* < 0.05). Presentation as in Fig. 2.

Analyses of the outer retina led to relatively concentric cluster patterns with a thickness bias towards the superior retina. Specifically, for the ONL_+HFL_, grid locations were assigned into five different clusters, with a slightly higher thickness profile superiorly (C_1_–C_4_) than inferiorly (C_2_–C_5_; Fig. 4A). All clusters were fitted with downward quadratic functions. C_F_ and C_1_ showed decline to begin at 40.6 and 25.8 years of age, respectively. However, slopes for C_2_–C_4_ suggested decline to begin outside of the ages within this study population (Fig. 4B,C). The rate of ONL_+HFL_ loss with age was not significantly different between all clusters (*p* = 0.92), and the pooled loss was calculated as − 0.06 µm/year.

Clustering analyses of the IS/OS led to assignment of three different clusters (Fig. 4D), with a slightly higher thickness profile superiorly (C_1_) compared to inferiorly (C_2_). All clusters were fitted with quadratic functions (Fig. 4E). Post-hoc analysis indicated that the rate of age-related IS/OS loss did not significantly differ between clusters (*p* = 0.28), and the pooled loss was − 0.04 µm/year; Fig. 4F).

Finally, for the RPE, seven different clusters were identified with a slightly higher thickness profile superiorly (C_1_–C_5_) and nasally (C_1_–C_5_) versus inferiorly (C_2_–C_6_) and temporally (C_2_–C_5_), respectively (Fig. 4G,H). As the RPE were not significantly associated with any variables including age, this suggested no age-related change in OPL thickness. This was confirmed in age regression analysis of all RPE cluster thicknesses (data not shown).

### Inter-layer comparisons

The comparison of slopes between the retinal layers showed no significant differences for the GCL, IPL, INL, and ONL_+HFL_ (*p* = 0.07, pooled loss =  − 0.07 µm/year). However, the IS/OS had a significantly lesser mean pooled slope (− 0.045 µm/year). Furthermore, the ages at which thickness began to decline was not significantly different between the GCL, IPL, INL, and ONL_+HFL_ (one-way ANOVA, *p* = 0.12; mean ± SD = 37.65 ± 6.79 years).

### Comparison between grid-wise clusters and ETDRS sectors

**Figure 5:**
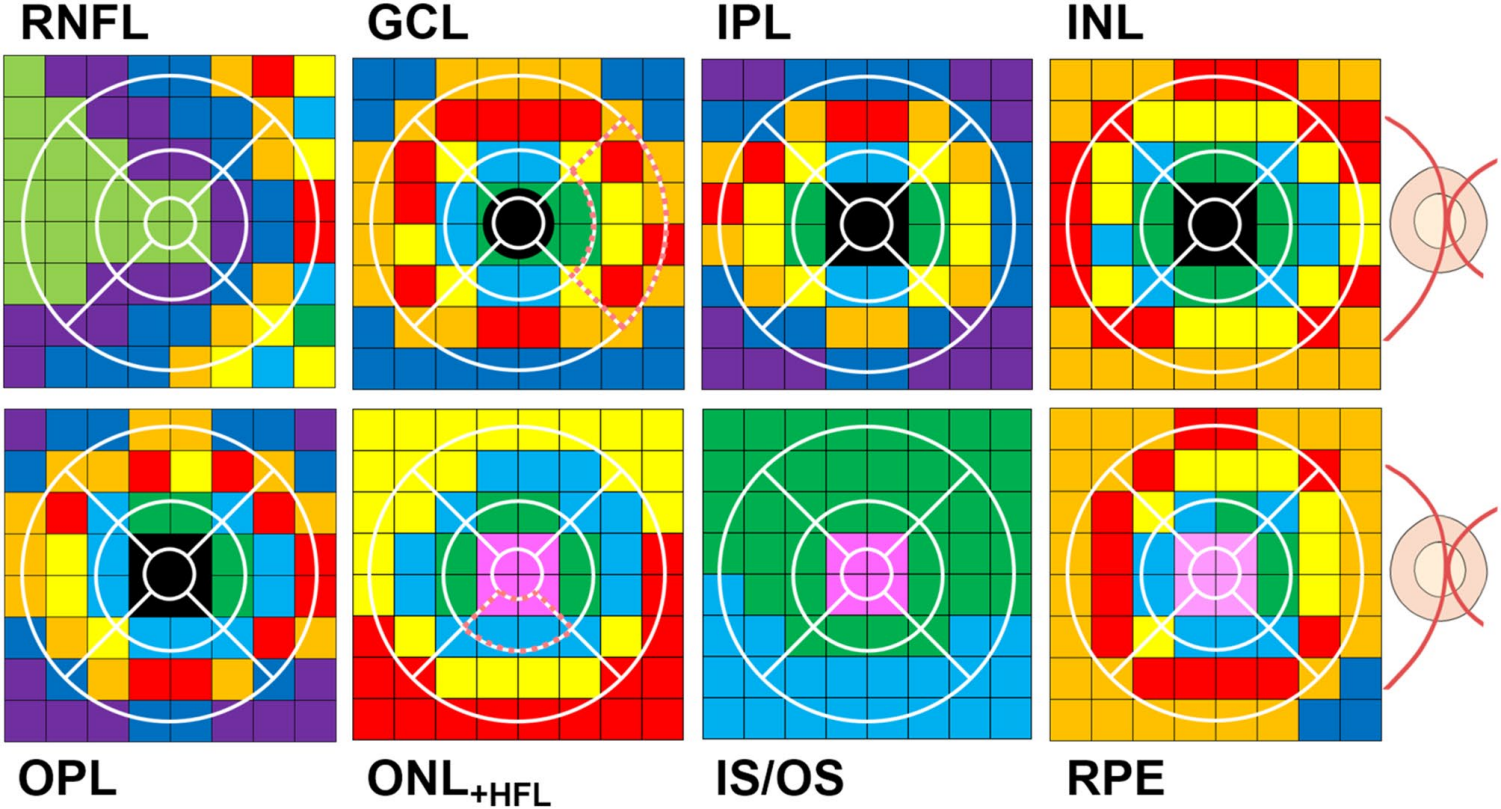
An illustrative comparison between spatial cluster topographies (in right eye format) and the ETDRS sectors. Note how there are multiple different clusters and hence multiple different average thicknesses and rates of age-related change within each ETDRS sector. Dotted markings highlight the GCL outer-ring nasal and ONL_+HFL_ inner-ring inferior ETDRS sectors, used for the quantitative examples below.

To demonstrate the advantage of retinal thickness analysis using grid-wise clusters over a more commonly used retinal spatial template, we overlayed the ETDRS sectors on spatial cluster topographies for each retinal layer (Fig. 5). In each retinal layer, almost every ETDRS sector contained multiple different clusters, and hence multiple different average thicknesses and rates of age-related change. Spatial discrepancies between the clusters and ETDRS sectors were particularly pronounced in the RNFL where the topography was not concentric.

To further demonstrate the improved accuracy of spatial clustering compared to ETDRS sector averaging, we provide quantitative examples whereby the averaged GCL outer-ring nasal and ONL_+HFL_ inner-ring inferior ETDRS sector thicknesses for each 10-yearly cohort interval were extracted (GCL, 10-yearly age interval cohorts from 20–29 to 70 + years, 38.79 ± 2.46, 38.85 ± 4.39, 38.86 ± 2.89, 37.83 ± 3.15, 36.68 ± 4.16, 34.17 ± 3.66 µm, respectively; ONL_+HFL_, 68.17 ± 9.82, 66.85 ± 11.91, 72.04 ± 7.87, 69.89 ± 9.19, 70.98 ± 10.17, 70.09 ± 8.62). These ETDRS sectors were selected as examples due to the dissimilar cluster composition, whereby the former contained four clusters (C_1_, C_3_, C_4_, and C_5_) and the latter contained only two clusters (C_F_, C_2_). The calculated coefficients of variation were then compared between the ETDRS sectors and the corresponding grid-wise clusters (Table 3). Subsequently, the combined grid-wise clusters demonstrated lower coefficients of variation compared to the ETDRS sectors (Mann–Whitney U test; GCL, clusters 0.05 ± 0.02, ETDRS sector 0.09 ± 0.02, *p* < 0.0001; ONL_+HFL_, clusters 0.03 ± 0.01, ETDRS sector 0.14 ± 0.02, *p* < 0.001).

**Table 3:** ETDRS sectors versus corresponding grid-wise clusters.

Cohort	ETDRS sector	C_1_	C_3_	C_4_	C_5_
**GCL outer-ring nasal**					
20–29	0.06	0.01	0.05	0.05	0.07
30–39	0.11	0.02	0.05	0.05	0.06
40–49	0.07	0.01	0.04	0.05	0.06
50–59	0.08	0.02	0.05	0.05	0.06
60–69	0.11	0.01	0.05	0.07	0.06
70+	0.11	0.04	0.05	0.05	0.07
Total	0.09 ± 0.02	0.05 ± 0.02			
Comparison	*p* < 0.0001				

Cohort	ETDRS sector	C_F_		C_2_	
**ONL**_**+HFL**_** inner-ring inferior**					
20–29	0.14	0.02		0.03	
30–39	0.18	0.02		0.03	
40–49	0.11	0.02		0.04	
50–59	0.13	0.02		0.04	
60–69	0.14	0.03		0.04	
70+	0.12	0.02		0.04	
Total	0.14 ± 0.02	0.03 ± 0.01			
Comparison	*p* < 0.001				

Values expressed are the coefficients of variation. Totals are expressed as mean ± standard deviation. Coefficients of variation for the grid-wise clusters are calculated from Supplementary Table 9.

ETDRS, Early Treatment for Diabetic Retinopathy Study; C_F_, _1…5_, foveal cluster and cluster 1 to 5.

## Discussion

Using location-specific cluster analysis of the Spectralis OCT 8 × 8 macular thickness grid, this study provides the spatial topography of each retinal layer. We found all layers exhibited concentric clusters surrounding the fovea except the RNFL which demonstrated an asymmetric radial pattern from the nasal retina. Our results validate previous spatial cluster methodologies used for the GCL^16^ and RNFL^23,24^ and demonstrate that these methodologies can be applied to other retinal layers. From these spatial clusters, we described non-linear rates of thickness decline in the majority of retinal layers, with similar rates of loss in the GCL, IPL, INL, and ONL_+HFL_, and lesser rates of thickness decline in the IS/OS. There were no age-related change in the RNFL, OPL, or RPE, and the RNFL were significantly associated with sex. These spatially defined normative data and regression functions provide an alternate and accessible method of retinal thickness analysis with more spatial detail and less variability compared to the ETDRS sectors, enabling more localised comparisons to structural and functional data of retinal and optic nerve disease.

### Spatial cluster topographies correspond to histological studies

Histological studies of the RNFL have consistently reported two spatial trends: greater thickness in the superior and inferior RNFL compared to the nasal and temporal RNFL; and decreasing RNFL thickness relative to distance from the optic nerve head^40–46^. These same trends were also evident in our cluster analysis, including a slight asymmetry whereby inferior clusters were slightly thicker than the superior clusters corresponding to some histological data^44^.

In the GCL, our study re-analysed data from Tong et al.^16^ using a different criterion and found similar clustering patterns, supporting the robustness of the spatial clustering approach. Previous histological studies have described a generally concentric ganglion cell density distribution decreasing towards the periphery, with a slight asymmetry between superior and nasal eccentricities compared to inferior and temporal eccentricities, respectively^47^. These trends were also seen in the GCL spatial cluster pattern in this study, with a slightly greater thickness profile in GCL clusters superiorly (C_4_, C_5_) and nasally (C_1_, C_3_, C_4_) compared to inferiorly (C_4_–C_6_) and temporally (C_2_–C_5_), respectively.

Validating the spatial clusters in other retinal layers is more challenging due to the scarcity of histological studies in humans. To our knowledge, only a single human study exists relating to INL topography, specifically focussed on bipolar cells. This study found a peak density along the temporal, horizontal meridian at 2–4 mm (7°–14°) from the fovea, with a subsequent decline towards the periphery^48^. Studies of primate eyes have suggested similar distributions for horizontal, amacrine, and Müller cells within the INL, with a central peak around 1 mm (5°)^49,50^ eccentricity and a slightly decreasing peripheral gradient^51^. The range of these peak densities are in agreement with our spatial cluster patterns for the INL and adjacent synaptic layers (the IPL and OPL), with peak thicknesses occurring at approximately 6° eccentricity in our study.

Finally, for the outer retina, histological studies have described cone peak density towards the foveal centre, which decreases rapidly to less than half at approximately 1.5 mm (5°) eccentricity (between the first and second concentrically arranged grids in our study)^52,53^. Similarly, rod peak density has been described around 4–5 mm (14°–17°) eccentricity, which decreases towards the periphery^48,54,55^. Some studies have also reported slightly higher rod peak density and S-cone density superiorly than inferiorly^55–57^. All the aforementioned studies would be in concordance with our ONL_+HFL_ and IS/OS spatial cluster patterns, showing peak thickness at the fovea with a decreasing peripheral gradient, particularly towards the inferior clusters. Meanwhile, our RPE spatial cluster pattern is also in agreement with studies that describe longer, more complex RPE microvilli/cone outer segment sheaths at the fovea, which then decrease in length and complexity as the population of cones decreases rapidly away from the fovea^58–60^, accompanied by a gradual peripheral decline in RPE cell density as well^61^.

### Rates of age-related thickness change from cluster analysis aligns with other studies

In the RNFL, we found no age-related change similar to other studies that have reported minimal to no age-related change in the macula RNFL^10–14^. It is important to note that while the RNFL does undergo an appreciable age-related decline as seen via histological and in vivo OCT studies, these studies are alternatively focussed on the peripapillary RNFL^5–9,45,62–65^.

In histological and OCT studies of the GCL, other studies have reported similar rates of age-related decline (− 0.05 to − 0.1 µm/year)^1,10–14,66,67^ in comparison to our study (− 0.03 to − 0.16 µm/year). Further discussions of the GCL spatial rates of change has already been expounded upon in Tong et al., whence the GCL data were obtained^16^.

Meanwhile, studies of the INL and the adjacent synaptic layers are scarce due to the complex arrangement of multiple distinct cellular components. Aggarwal et al.^2^ demonstrated an approximately − 0.7%/year decline in rod bipolar cells after 35 years of age, however, this value was derived from only four histological human samples and is incomparable to our figures, as our OCT analysis of the INL is indiscriminant of cell type. Another histological study of primate eyes has also demonstrated an age-related decline in rod bipolar, horizontal, and amacrine cell populations, although exact values were not provided^68^. Age-related decline of the IPL, INL, and OPL has been quantified more so in OCT studies, with similar rates of IPL and INL thinning (IPL − 0.03 to − 0.07 µm/year; INL − 0.03 to − 0.04 µm/year) , and relatively stable OPL thickness with age as compared to our study (pooled slopes for the IPL and INL, − 0.1 µm/year and − 0.05 µm/year; and no age-related change in OPL thickness)^10–14,17^.

Finally, regarding the outer retina, the ONL_+HFL_ and IS/OS in our study showed similar trends of age-related decline (pooled slopes for the ONL_+HFL_ and IS/OS, − 0.06 µm/year and − 0.04 µm/year, respectively) when compared to both histological and OCT studies reporting significant rod loss^3,4,10,12,14,17,69^, and minimal cone loss with age^3,4,67^. In our study, this was highlighted by the lesser change at the central foveal cluster of the ONL_+HFL_, which contains a significantly higher proportion of cones to rods than elsewhere. In the RPE, histological studies have demonstrated age-related decline up to approximately − 0.3%/year^61,67^. In this study, we however observed no significant age-related change in RPE thickness. Other OCT studies have reported conflicting trends^10–12,14^. These discrepancies are likely due to OCT’s inability to distinguish the reflectance profiles of RPE cells, melanin, lipofuscin, and adjacent structures such as basal deposits that may ‘fill in’ space with age^10–12,14,60,70^, potentially masking RPE thickness decline using OCT.

### Retinal layers display inter-connected rates of age-related decline

Across individual retinal layers, age-related thickness changes were variable when considered in raw units, i.e. µm. However, further comparison of these values to their location-specific peak thickness (at the vertex point) resulted in similarities within and between most individual retinal layers. The majority of regression models suggested preservation of the thickness of these layers until approximately the late 4th decade of age, followed by a steady decline. This is consistent with previous studies that also demonstrate relative preservation of the ganglion cells, bipolar cells, and photoreceptor cell bodies until a similar age^2,3,16,71^.

The exception to this was the RNFL, OPL, IS/OS, and RPE. The RNFL demonstrated significant association between sex and thickness, but no significant association with age were found. Greater RNFL thickness in females compared to males as we found (albeit not reaching statistical significance for location-specific cluster analysis) were in accordance with other studies^12,72–75^, explained by the potential neuroprotective effects of estrogen^76–78^.

For the OPL, there were also no significant age-related change. This was surprising considering the other synaptic layer—the IPL—demonstrated similar trends of decline to its adjacent neuronal layers. Lack of histological topographical studies regarding the synaptic layers in human retina make it difficult to determine the reason for these effects, but it is possible that the displacement of nuclei from the adjacent ONL, which increases considerably after age forty^79^, offsets the otherwise expected decrease in thickness around the same age. Another possibility is that in ageing, horizontal and rod-bipolar cell dendrites may ‘sprout’ within the OPL^80,81^, subsequently offsetting the expected decrease in thickness as well. Nonetheless, what truly happens to the OPL with age is currently speculative, as anatomical changes in the OPL may be masked or skewed by the adjacent segmentation of the ONL_+HFL_, as defined by OCT.

In the IS/OS, age-related thickness decline was less than all other retinal layers except the OPL and RPE. Histological studies have shown plasticity in the ageing rod system, whereby decreasing populations of rod cells are compensated for by enlargement of the remaining rod inner segments, resulting in an overall similar area of coverage^3,4^. Additionally, photoreceptor outer segments have shown increased axial thickness in OCT studies^13,82,83^, possibly reflecting the age-related outer segment nodular swelling and disorganisation as seen in histological studies^71,84^. These effects are thought to be associated with reduced support from a declining retinal pigment epithelium population in ageing^61,70^.

Finally, in the RPE, there was no age-related thickness decline. As aforementioned, this may be an artefact of the OCT technology’s inability to differentiate RPE cells, melanin, lipofuscin, and other adjacent structures that contribute to its reflectance pattern. Or, it may reflect the increase in size and disorganisation of RPE cells with age, as well as the migration of more peripheral RPE cells to compensate for macula RPE cell death, offsetting OCT thickness decline to an extent^85^.

### Clustering of grid-wise thickness data compared to ETDRS sector averaging

In all individual retinal layers, we demonstrated that multiple clusters (and hence multiple different average thicknesses and rates of age-related change) exist within each ETDRS sector. This suggests that the averaging of thickness data based on ETDRS sectors may produce inaccurate outcomes, particularly when attempting to compare more localised lesions to normative data. We confirmed this in our quantitative examples, whereby the grid-wise cluster approach demonstrated reduced variability and hence improved ability to identify localised thickness differences compared to the ETDRS sectoral approach. Use of the grid-wise cluster approach is even more valuable when considering that a previous study of diseased eyes revealed disparate changes (i.e. GCL thinning and thickening) between clusters of the GCL which otherwise has not previously been reported via OCT^21^, likely due to the relatively large spatial averaging of the ETDRS sectors. Overall, while our grid-wise cluster approach shows superiority over the currently used ETDRS sectoral approach, it may be possible to develop OCT thickness spatial analyses with even greater detail using customised data extraction methods. Of course, this method is not easily accessible compared to the commercially available 8 × 8 grid or ETDRS sectors in the Spectralis SD-OCT. However, it would more clearly delineate junctional zones between the spatial cluster patterns that we have presented, as well as define areas at the foveal slope which we could not accurately quantify due to the high variability of thicknesses. Current work is underway to validate the higher-density sampling method of OCT thickness spatial analysis^86^.

## Limitations

This study was associated with some limitations. Firstly, due to the extensive range of ages included in our study, we measured retinal age-related thickness changes using cross-sectional, rather than longitudinal data. Our use of strict inclusion criteria, large sample size, and the clustering of grid-wise data, however, helped to mitigate potential confounding co-variables and inter-individual variabilities that may be more prevalent in cross-sectional data. In addition, our regression functions enable the potential age-correction of individual retinal layer thickness data, thus improving statistical power of future studies that may also use cross-sectional cohorts instead of longitudinal cohorts as well. This method has been previously validated in analysis of GCL thickness in diseased and normal eyes, and current work to validate the use of age-correction functions in other retinal layers is underway^21^.

Secondly, as this was a retrospective, cross-sectional study, age-related changes were sourced from cohorts of 23–69 eyes per decade from the total cohort of 253 eyes. Cross-sectional data by design may present with confounding factors between unpaired groups that are less easily controlled for than longitudinal data with paired groups. However, our initial multi-variable analysis demonstrated that only age and sex were significant variables affecting outcome thicknesses in certain layers, which were then accounted for by grouping our cohort by age and sex in the appropriate layers. Additionally, our study still fulfils definitions for establishing normative data^87^ including: (1) precise characterisation of the study population via multi-variable analysis to determine variables associated with thickness outcomes; (2) clear definition and measurement of phenomena, i.e. spatial topography and rates of age-related thickness change for each retinal layer; and (3) appropriate interpretion and generalisation of results in the context of other OCT and histological studies. Additionally, our sample size is similar, if not higher compared to many other recent normative databases for the Spectralis SD-OCT ranging from n = 50–297^28–32^.

Finally, as OCT is essentially an analysis of reflectance profiles, changes in retinal thickness do not confer information about which specific cellular structures are affected. This limitation was significant in the RNFL, as the main retinal arterioles and venules cause significant variability in thickness measurements^88^ and may explain why we, along with another histological study, found the RNFL to be thickest inferiorly^44^ as opposed to other studies which found the RNFL to be thickest superiorly^40–43^. The ambiguity of reflectance profiles in OCT is also evident in analysis of Henle’s fibre layer which we kept as part of the ONL segmentation to maintain consistency with other studies and OCT auto-segmentation protocol, despite anatomically being part of the OPL. Furthermore, the inability to separate the photoreceptor IS from the OS with the current HRA/Spectralis Viewing Module limits interpretation of changes in these layers. Current work is underway to collate vascular parameters and retinal layer thickness changes in normal ageing, via OCT-angiography and OCT respectively. Future software updates could also rectify OCT segmentation limitations.

## Conclusion

Individual retinal layer thicknesses demonstrate specific topographical patterns and similar rates of normal, age-related change. Specifically, RNFL thickness displayed an outwards, radial pattern from the nasal retina, while all other layers from the GCL to the RPE displayed concentric patterns of thickness decreasing from the fovea. Similar rates of age-related thickness change were observed in the GCL, IPL, INL, and ONL_+HFL_, while the IS/OS demonstrated lesser rates of thickness decline. There were no age-related change in the RNFL, OPL, or RPE, and the RNFL were significantly associated with sex. From these grid-wise clustered data, we have established a spatially defined normative database alongside age-correction functions. These provide an accessible method of retinal thickness analysis with more spatial detail and less variability than the ETDRS sectors, to aid the diagnosis and monitoring of retinal and optic nerve disease.

## Supplementary Information

Supplementary Tables.

## Data Availability

The datasets generated during and/or analysed during the current study are available from the corresponding author upon reasonable request.
