# Supplementary material for: Modelling normal age-related changes in individual retinal layers using location-specific OCT analysis
Source: Sci Rep. 2021 Jan 12;11:558. doi: 10.1038/s41598-020-79424-6 (PMC7804110; doi:10.1038/s41598-020-79424-6)

**Supplementary material**

**Modelling normal age-related changes in individual retinal layers using location-specific OCT analysis**

Matt Trinh^1,2^, Vincent Khou^1,2^, Barbara Zangerl^1,2^, Michael Kalloniatis^1,2^, and Lisa Nivison-Smith^1,2,*^

*^1^ Centre for Eye Health, University of New South Wales, Sydney, 2052, Australia; ^2^ School of Optometry and Vision Science, University of New South Wales, Sydney, 2052, Australia*

***Correspondence:**

Dr Lisa Nivison-Smith

School of Optometry and Vision Science, UNSW Australia, Sydney, 2052, NSW, Australia.

Phone: Int +61 2 81150791

Fax: Int +61 2 81150799

E-mail: [l.nivison-smith@unsw.edu.au](mailto:l.nivison-smith@unsw.edu.au)

**Tables S1-8.**

Each table below represents an individual retinal layer and describes the resultant cluster patterns using hierarchical and k-means clustering. For each retinal layer, input data for clustering were grouped according to variables that were significantly associated with average thickness. Specifically: the RNFL is clustered by sex; the GCL, IPL, INL, ONL, and IS/OS are clustered by age cohorts; and the OPL and RPE are clustered with singular grouping using the whole cohort. Selection of the final cluster pattern (**bolded**, *italicised*, and underlined) were based off the optimal regression fit (R^2^, sum-of-squares, and SD-of-residuals) where available, or lowest coefficient of variation.

Abbreviations: C_1-8_, cluster_1-8_.

**Table S1. Characteristics for the derived RNFL cluster patterns**

Input data for clustering were grouped according to sex.

| **RNFL clustering method** | **Coefficient of variation** |
| --- | --- |
| Hierarchical  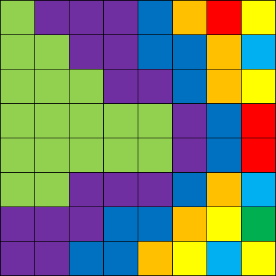**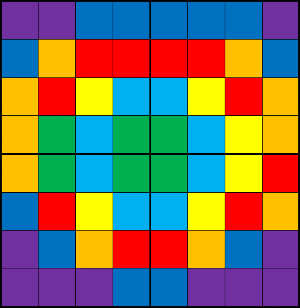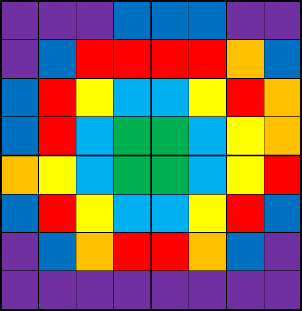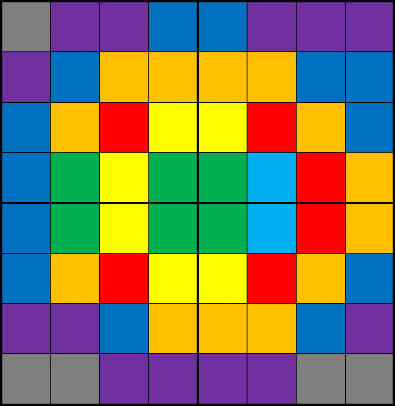**C_1_  C_2_  C_3_  C_4_  C_5_  C_6_  C_7_  C_8_  Mean | 0  0.06  0.05  0.05  0.07  0.09  0.14  0.36  0.1 |
| ***K-means***  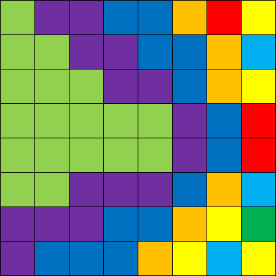**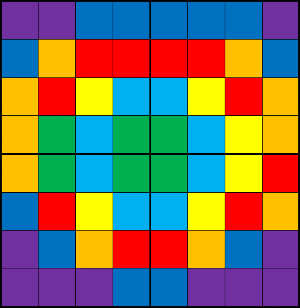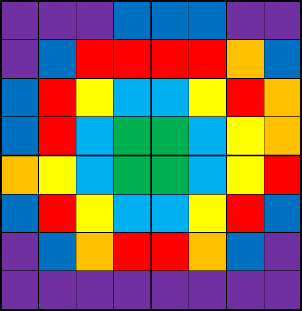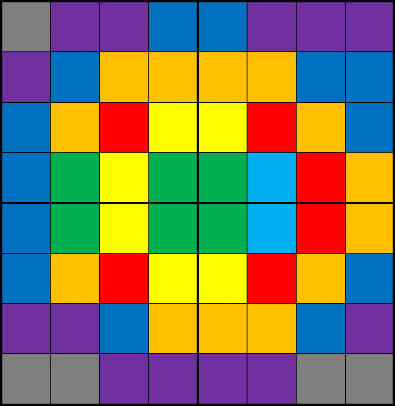**C_1_  C_2_  C_3_  C_4_  C_5_  C_6_  C_7_  C_8_  Mean | 0  0.06  0.05  0.05  0.07  0.11  0.12  0.12  0.07 |
| Mann Whitney *U*-test  (p-value) | 0.91 |

**Table S2. Characteristics for the derived GCL cluster patterns**

Input data for clustering were grouped according to age cohorts.

| **GCL clustering method** | **R^2^** | **Sum-of-squares** | **SD-of-residuals** |
| --- | --- | --- | --- |
| Hierarchical 5yearly  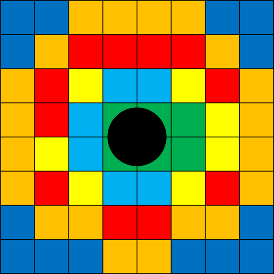**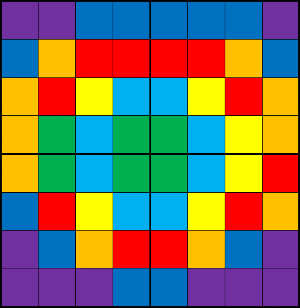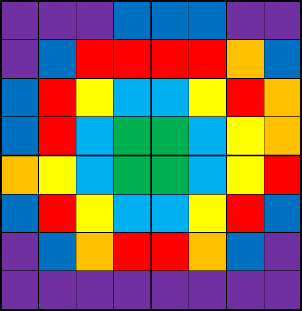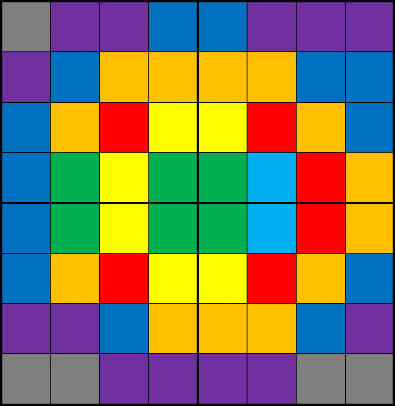**C_1_  C_2_  C_3_  C_4_  C_5_  C_6_  Mean | 0.77  0.91  0.9  0.86  0.79  0.5  0.79 | 5.3  2.61  2.41  2.04  1.36  1.31  2.51 | 0.81  0.57  0.55  0.51  0.41  0.4  0.54 |
| K-means 5yearly  C_1_  C_2_  C_3_  C_4_  C_5_  C_6_  Mean | 0.77  0.91  0.9  0.88  0.77  0.52  0.79 | 5.3  2.61  2.41  1.75  1.55  1.26  2.48 | 0.81  0.57  0.55  0.47  0.44  0.4  0.54 |
| Hierarchical 10yearly  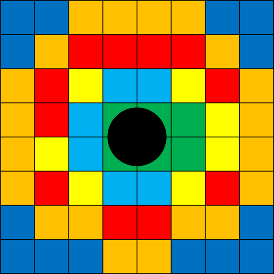C_1_  C_2_  C_3_  C_4_  C_5_  C_6_  Mean | 0.86  0.98  0.98  0.94  0.91  0.64  0.89 | 2.21  0.42  0.34  0.54  0.39  0.6  0.75 | 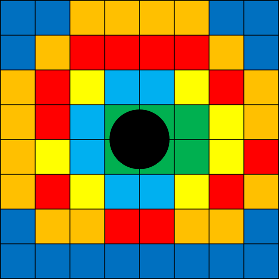  0.86  0.37  0.33  0.42  0.36  0.45  0.47 |
| ***K-means 10yearly***  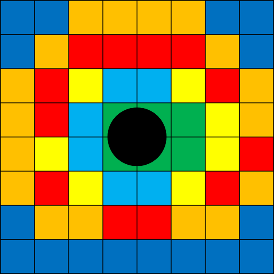C_1_  C_2_  C_3_  C_4_  C_5_  C_6_  Mean | 0.86  0.98  0.98  0.96  0.89  0.66  0.89 | 2.21  0.42  0.34  0.4  0.5  0.54  0.74 | 0.86  0.37  0.33  0.37  0.41  0.43  0.46 |
| Kruskal-Wallis comparison (p-values) | 0.22 | **< 0.01** | 0.24 |

**Table S3. Characteristics for the derived IPL cluster patterns**

Input data for clustering were grouped according to age cohorts.

| **IPL clustering method** | **R^2^** | **Sum-of-squares** | **SD-of-residuals** |
| --- | --- | --- | --- |
| Hierarchical 5yearly  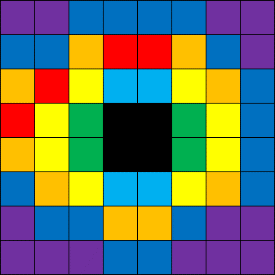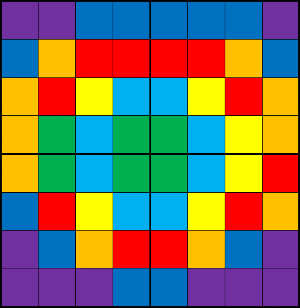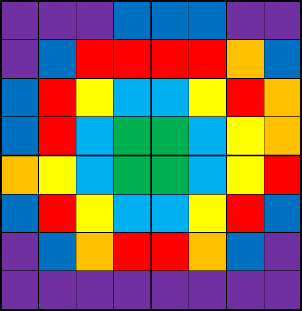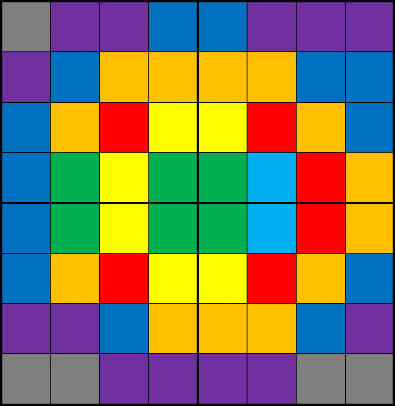C_1_  C_2_  C_3_  C_4_  C_5_  C_6_  C_7_  Mean | 0.81  0.9  0.7  0.88  0.55  0.18  0.13  0.59 | 2.15  1.29  3.26  0.85  2.7  4.44  6.49  3.03 | 0.52  0.4  0.64  0.33  0.58  0.74  0.9  0.59 |
| K-means 5yearly  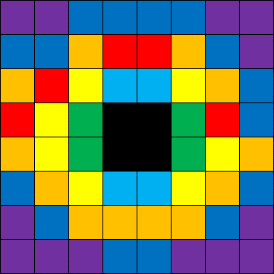C_1_  C_2_  C_3_  C_4_  C_5_  C_6_  C_7_  Mean | 0.81  0.9  0.7  0.84  0.5  0.13  0.14  0.57 | 2.15  1.29  2.84  1.39  2.97  4.67  6.33  3.09 | 0.52  0.4  0.6  0.42  0.61  0.76  0.89  0.6 |
| ***Hierarchical 10yearly***  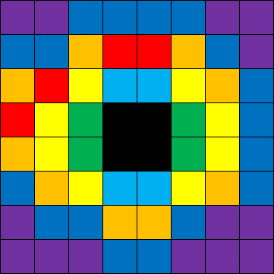C_1_  C_2_  C_3_  C_4_  C_5_  C_6_  C_7_  Mean | 0.92  0.95  0.83  0.97  0.69  0.36  0.14  0.69 | 0.69  0.43  1.38  0.14  1.3  2.4  3.43  1.4 | 0.48  0.38  0.68  0.22  0.66  0.89  1.07  0.63 |
| K-means 10yearly  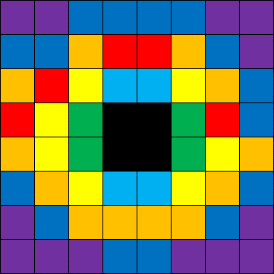C_1_  C_2_  C_3_  C_4_  C_5_  C_6_  C_7_  Mean | 0.92  0.95  0.83  0.93  0.64  0.3  0.15  0.67 | 0.69  0.43  1.2  0.44  1.54  2.52  3.35  1.45 | 0.48  0.38  0.63  0.38  0.72  0.92  1.06  0.65 |
| Kruskal-Wallis comparison (p-values) | 0.55 | 0.09 | 0.99 |

**Table S4. Characteristics for the derived INL cluster patterns**

Input data for clustering were grouped according to age cohorts.

| **INL clustering method** | **R^2^** | **Sum-of-squares** | **SD-of-residuals** |
| --- | --- | --- | --- |
| Hierarchical 5yearly  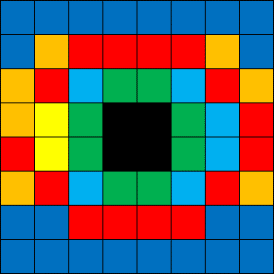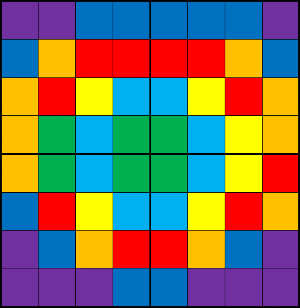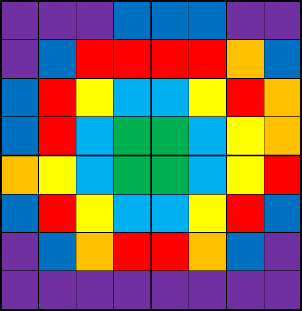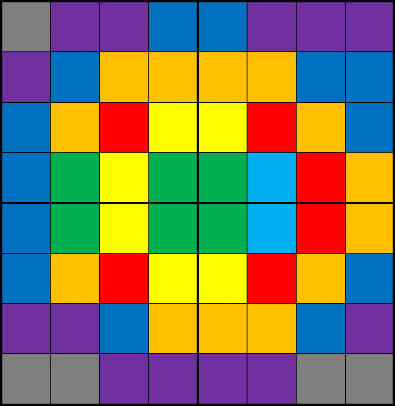C_1_  C_2_  C_3_  C_4_  C_5_  C_6_  Mean | 0.39  0.72  0.44  0.77  0.83  0.79  0.66 | 3.38  3.3  5.5  1.9  1.08  1.36  2.75 | 0.65  0.64  0.83  0.49  0.37  0.41  0.57 |
| K-means 5yearly  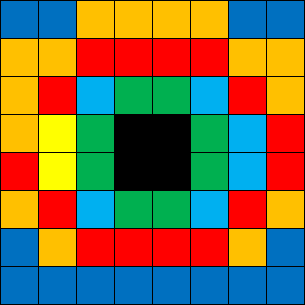C_1_  C_2_  C_3_  C_4_  C_5_  C_6_  Mean | 0.39  0.72  0.44  0.77  0.84  0.72  0.65 | 3.38  3.3  5.5  1.79  1.04  1.77  2.8 | 0.65  0.64  0.83  0.47  0.36  0.47  0.57 |
| Hierarchical 10yearly  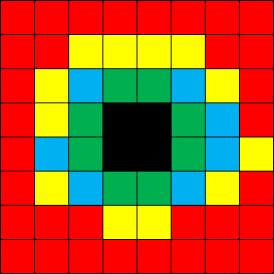C_1_  C_2_  C_3_  C_4_  _-_  _-_  Mean | 0.62  0.86  0.91  0.96  0.84 | 1.41  0.97  0.44  0.14  0.74 | 0.69  0.57  0.38  0.22  0.47 |
| ***K-means 10yearly***  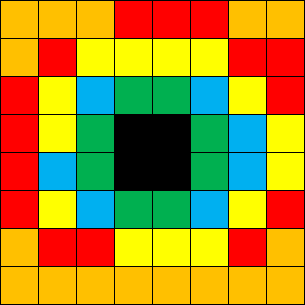C_1_  C_2_  C_3_  C_4_  C_5_  -  Mean | 0.62  0.86  0.92  0.98  0.85  0.85 | 1.41  0.97  0.41  0.05  0.45  0.66 | 0.69  0.57  0.37  0.13  0.39  0.43 |
| Kruskal-Wallis comparison (p-values) | 0.06 | **< 0.01** | 0.69 |

**Table S5. Characteristics for the derived OPL cluster patterns**

Input data for clustering were a singular group.

| **OPL clustering method** | **Coefficient of variation** |
| --- | --- |
| ***Hierarchical***  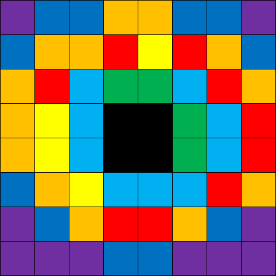**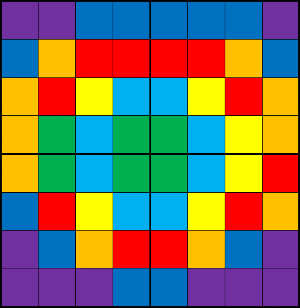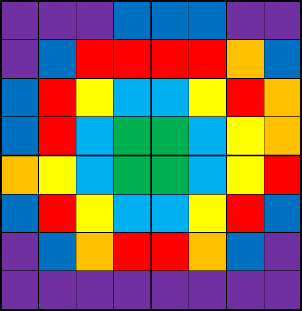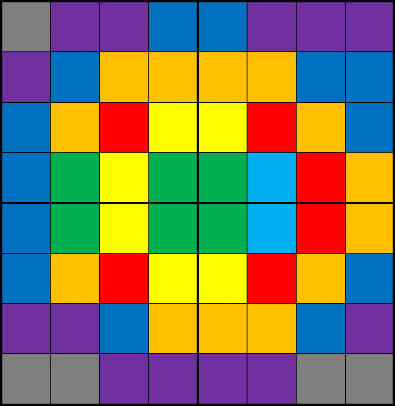**C_1_  C_2_  C_3_  C_4_  C_5_  C_6_  C_7_  Mean | 0.01  0.01  0.01  0.01  0.02  0.01  0.01  0.01 |
| K-means  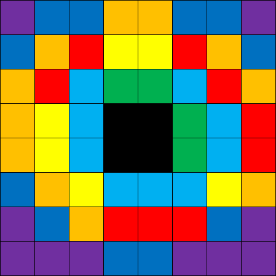**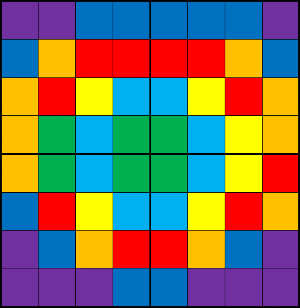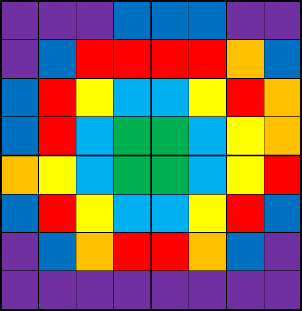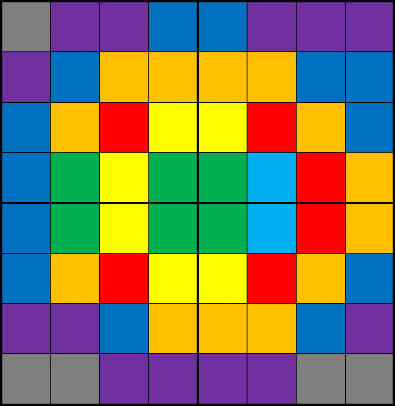**C_1_  C_2_  C_3_  C_4_  C_5_  C_6_  C_7_  Mean | 0.01  0.01  0.02  0.02  0.01  0.01  0.01  0.01 |
| Mann Whitney *U*-test  (p-value) | 0.44 |

**Table S6. Characteristics for the derived ONL_+HFL_ cluster patterns**

Input data for clustering were grouped according to age cohorts.

| **ONL_+HFL_**  **clustering method** | **R^2^** | **Sum-of-squares** | **SD-of-residuals** |
| --- | --- | --- | --- |
| Hierarchical 5yearly  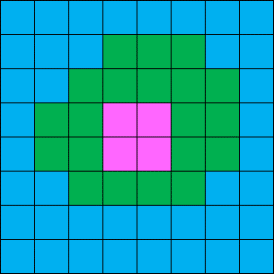C_F_  **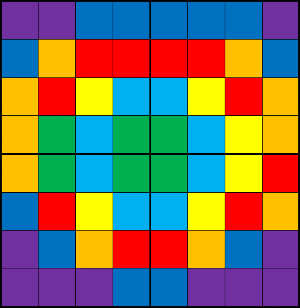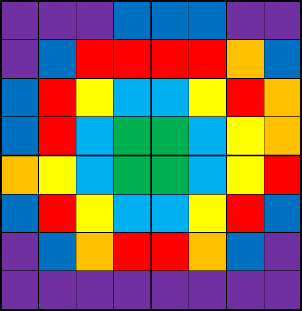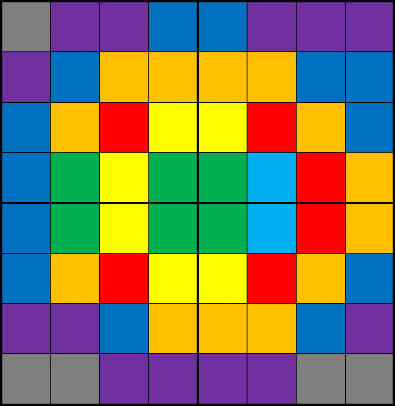**C_1_  C_2_  -  -  -  Mean | 0.34  0.62  0.54  0.5 | 6.27  6.31  9.04  7.21 | 0.89  0.89  1.06  0.95 |
| K-means 5yearly  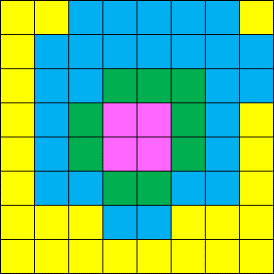C_F_  **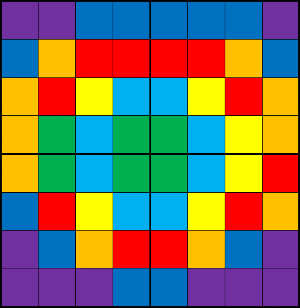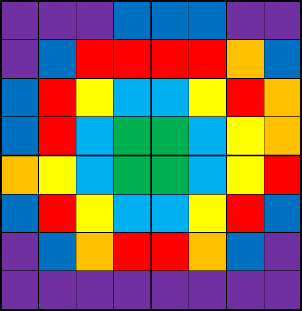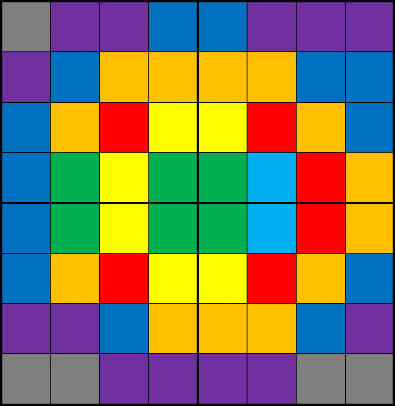**C_1_  C_2_  C_3_  -  -  Mean | 0.34  0.61  0.6  0.51  0.52 | 6.27  5.39  8.11  9.4  7.29 | 0.89  0.82  1.01  1.08  0.95 |
| Hierarchical 10yearly  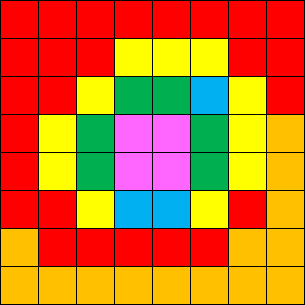C_F_  **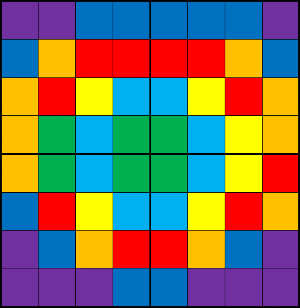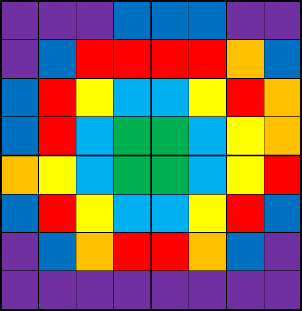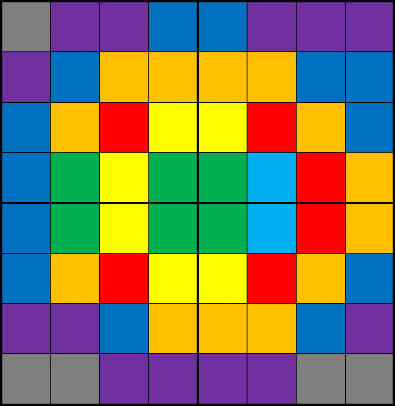**C_1_  C_2_  C_3_  C_4_  C_5_  Mean | 0.83  0.94  0.91  0.94  0.93  0.91  0.91 | 0.35  0.37  0.43  0.48  0.49  0.55  0.45 | 0.34  0.35  0.38  0.4  0.4  0.43  0.38 |
| ***K-means 10yearly***  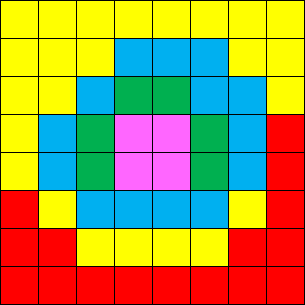C_F_  **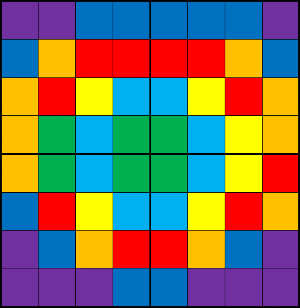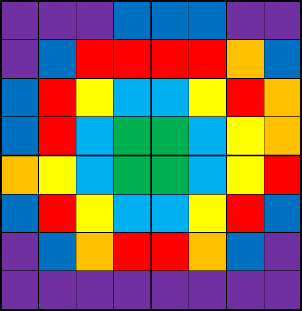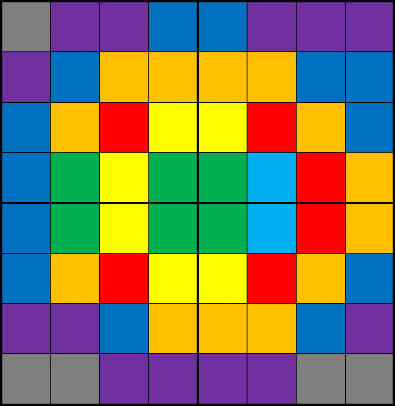**C_1_  C_2_  C_3_  C_4_  -  Mean | 0.83  0.94  0.94  0.93  0.92  0.91 | 0.35  0.37  0.45  0.51  0.49  0.43 | 0.34  0.35  0.39  0.41  0.41  0.38 |
| Kruskal-Wallis comparison (p-values) | **<0.001** | **< 0.001** | **< 0.001** |

**Table S7. Characteristics for the derived IS/OS cluster patterns**

Input data for clustering were grouped according to age cohorts.

| **IS/OS clustering method** | **R^2^** | **Sum-of-squares** | **SD-of-residuals** |
| --- | --- | --- | --- |
| Hierarchical 5yearly  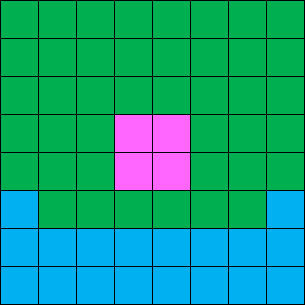C_F_  **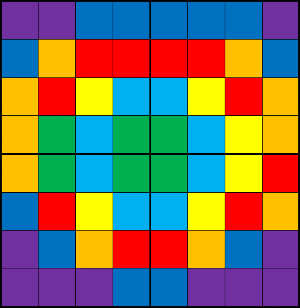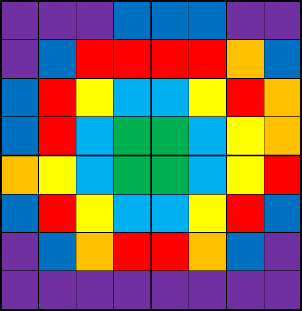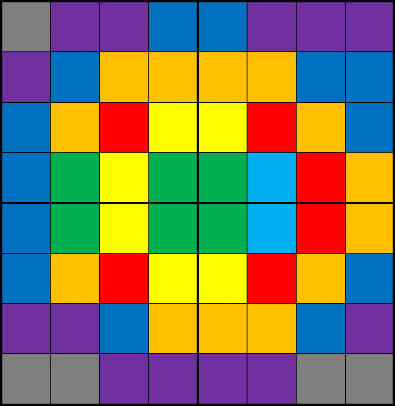**C_1_  C_2_  -  -  -  Mean | 0.7  0.79  0.28  0.59 | 3.4  0.7  0.94  1.68 | 0.65  0.3  0.34  0.43 |
| K-means 5yearly  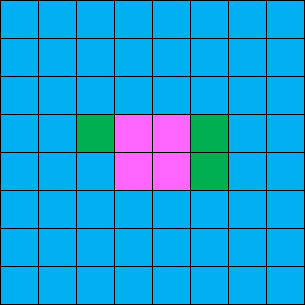C_F_  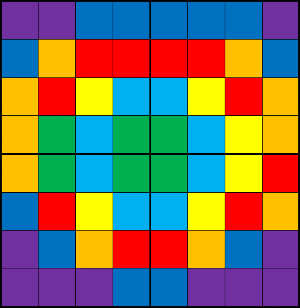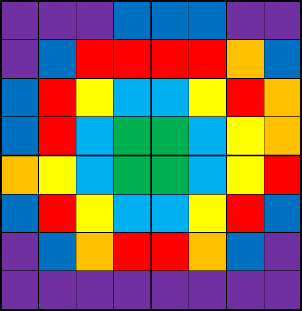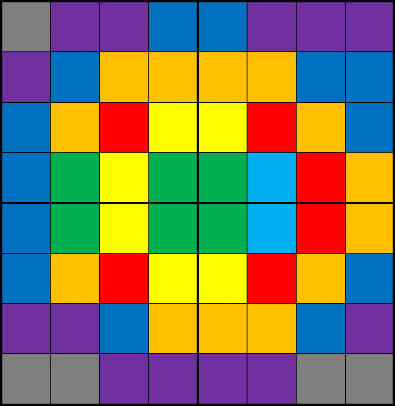C_1_  C_2_  -  -  -  Mean | 0.7  0.86  0.7  0.75 | 3.4  1.38  0.63  1.8 | 0.65  0.41  0.28  0.45 |
| Hierarchical 10yearly  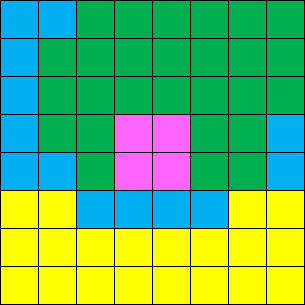C_F_  **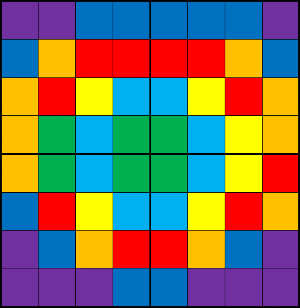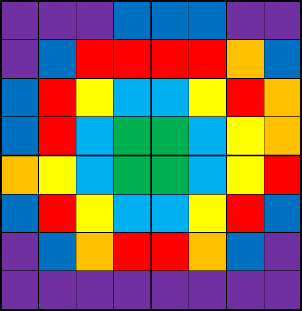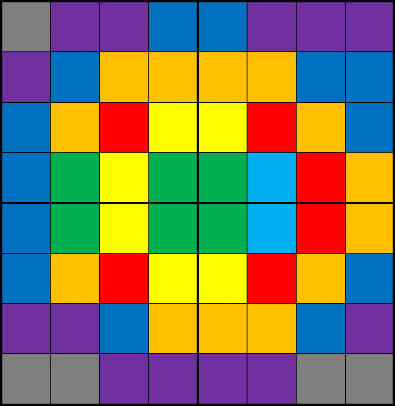**C_1_  C_2_  C_3_  -  -  Mean | 0.86  0.85  0.88  0.79  0.85 | 0.77  0.29  0.18  0.17  0.35 | 0.51  0.31  0.24  0.15  0.3 |
| ***K-means 10yearly***  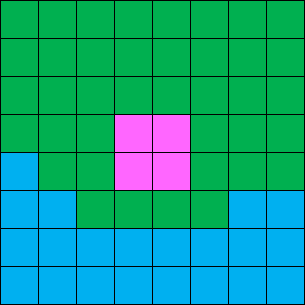C_F_  **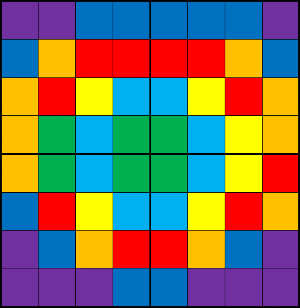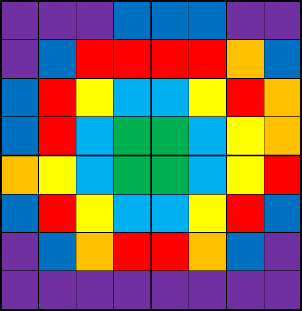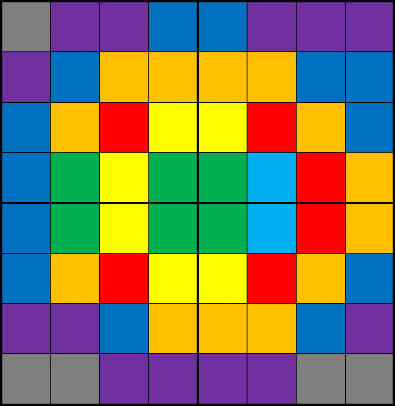**C_1_  C_2_  -  -  -  Mean | 0.86  0.86  0.79  0.84 | 0.67  0.24  0.07  0.33 | 0.51  0.28  0.15  0.31 |
| Kruskal-Wallis comparison (p-values) | 0.13 | 0.06 | 0.56 |

**Table S8. Characteristics for the derived RPE cluster patterns**

Input data for clustering were a singular group.

| **RPE clustering method** | **Coefficient of variation** |
| --- | --- |
| ***Hierarchical***  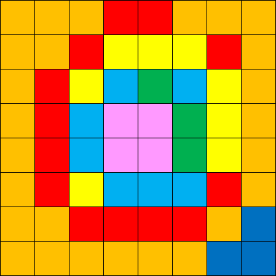C_F_  **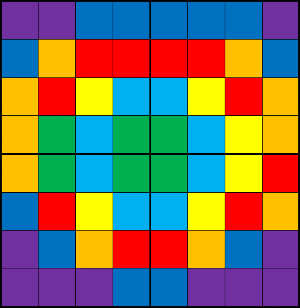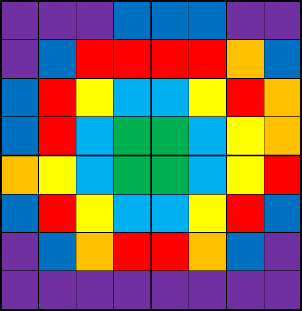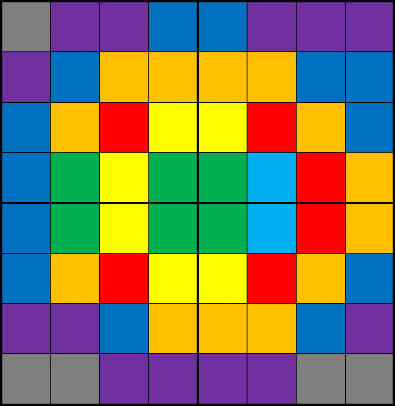**C_1_  C_2_  C_3_  C_4_  C_5_  C_6_  Mean | 0.03  0.01  0.01  0.01  0.01  0.01  0.01  0.01 |
| K-means  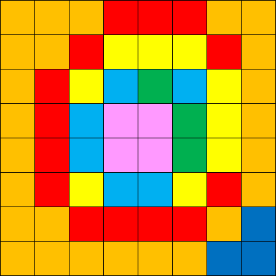C_F_  **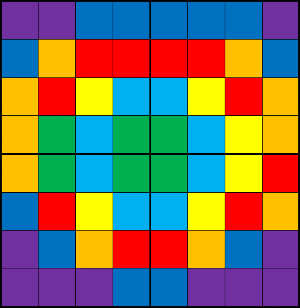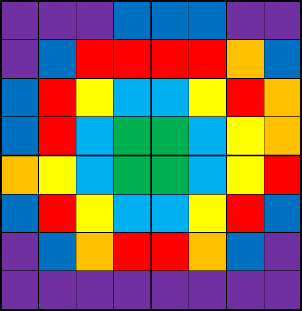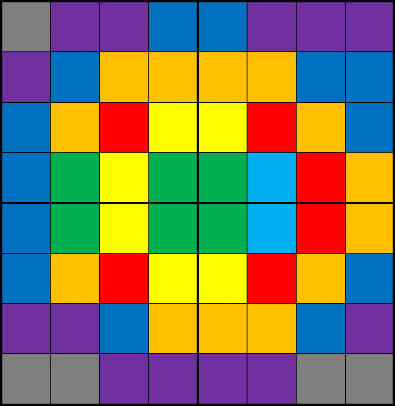**C_1_  C_2_  C_3_  C_4_  C_5_  C_6_  Mean | 0.03  0.01  0.01  0.01  0.01  0.01  0.01  0.01 |
| Mann Whitney *U*-test  (p-value) | 0.73 |

**Table S9. Normative data**

Average cluster thickness (µm ± SD) for each retinal layer, grouped by significant variable (derived from a multi-variable model including age, sex, ethnicity, spherical equivalent refraction, and BCVA versus average retinal thickness). Hence the RNFL was clustered by sex; the GCL, IPL, INL, ONL, and IS/OS were clustered by age cohorts; and the OPL and RPE were clustered as a singular group. For reference, the final cluster patterns (right eye format) for each retinal layer are included *below*.

|  | | **Grouping** | | | | | |
| --- | --- | --- | --- | --- | --- | --- | --- |
| **RNFL** |  | **Female** | | | **Male** | | |
|  | C_1_ | 122.27 (0) | | | 121.78 (0) | | |
|  | C_2_ | 105.56 (7.52) | | | 100.83 (3.74) | | |
|  | C_3_ | 91.96 (3.39) | | | 88.17 (5.03) | | |
|  | C_4_ | 75.84 (3.93) | | | 72.75 (3.95) | | |
|  | C_5_ | 63.55 (4.3) | | | 58.38 (3.3) | | |
|  | C_6_ | 44.7 (4.94) | | | 42.32 (4.12) | | |
|  | C_7_ | 30.13 (3.65) | | | 29.32 (3.44) | | |
|  | C_8_ | 18.55 (2.36) | | | 18.58 (2.26) | | |
| **GCL** |  | **20-29 years** | **30-39 years** | **40-49 years** | **50-59 years** | **60-69 years** | **70+ years** |
|  | C_1_ | 56.84 (0.63) | 56.05 (1.02) | 56.26 (0.6) | 57.06 (0.89) | 55.21 (0.57) | 52.23 (2.22) |
|  | C_2_ | 51.13 (1.88) | 52.01 (1.16) | 51.46 (1.4) | 51.24 (1.53) | 50.17 (1.04) | 46.74 (1.67) |
|  | C_3_ | 42.84 (1.96) | 43.3 (1.96) | 42.99 (1.91) | 42.05 (2.01) | 41.48 (1.91) | 38.31 (2.11) |
|  | C_4_ | 33.36 (1.79) | 34.21 (1.74) | 33.71 (1.76) | 32.91 (1.62) | 32.65 (2.29) | 30.24 (1.41) |
|  | C_5_ | 26.6 (1.77) | 27.32 (1.71) | 27.05 (1.65) | 26.33 (1.68) | 26.56 (1.62) | 24.6 (1.62) |
|  | C_6_ | 22.18 (1.35) | 22.9 (1.34) | 22.27 (1.29) | 21.9 (1.19) | 22.34 (1.02) | 21.18 (1.26) |
| **IPL** |  | **20-29 years** | **30-39 years** | **40-49 years** | **50-59 years** | **60-69 years** | **70+ years** |
|  | C_1_ | 43.22 (0.51) | 42.89 (0.52) | 43.22 (0.55) | 42.94 (0.42) | 42.46 (0.27) | 39.8 (0.96) |
|  | C_2_ | 40.3 (0.05) | 40.11 (0.49) | 39.9 (0.2) | 39.67 (0.29) | 39.11 (0.12) | 36.64 (0.15) |
|  | C_3_ | 34.02 (1.21) | 34 (0.84) | 33.96 (1.32) | 33.43 (1.45) | 33.87 (0.96) | 30.77 (1.88) |
|  | C_4_ | 29.97 (0.3) | 30.07 (0.72) | 29.96 (0.35) | 29.42 (0.4) | 29.24 (0.54) | 27.57 (0.96) |
|  | C_5_ | 26.78 (0.86) | 26.94 (0.88) | 26.64 (1.08) | 26.11 (1.08) | 26.96 (0.9) | 24.56 (1.17) |
|  | C_6_ | 21.88 (1.6) | 22.22 (1.62) | 21.48 (1.6) | 21.25 (1.65) | 22.66 (1.4) | 20.18 (1.3) |
|  | C_7_ | 17.23 (1.16) | 17.83 (1.27) | 17.48 (0.99) | 17.3 (1.13) | 19.47 (0.86) | 17.06 (0.91) |
| **INL** |  | **20-29 years** | **30-39 years** | **40-49 years** | **50-59 years** | **60-69 years** | **70+ years** |
|  | C_1_ | 39.93 (0.99) | 41.24 (1.12) | 40.24 (0.93) | 40.32 (1.13) | 40.82 (1.22) | 38.74 (1.05) |
|  | C_2_ | 36.84 (1.12) | 37.57 (1.27) | 36.51 (0.91) | 36.2 (0.77) | 36.36 (0.91) | 34.01 (0.82) |
|  | C_3_ | 31.58 (0.81) | 32.05 (1.06) | 31.2 (1.21) | 30.82 (1.08) | 30.76 (1.59) | 29.04 (0.86) |
|  | C_4_ | 27.91 (1.04) | 28.04 (1.13) | 27.59 (0.99) | 27.16 (1.09) | 26.88 (1.42) | 25.86 (1.05) |
|  | C_5_ | 24.77 (1.09) | 25.15 (1.02) | 24.47 (1.03) | 24.14 (0.87) | 23.24 (1.19) | 23.35 (0.95) |

**INL**

**IPL**

**GCL**

**RNFL**


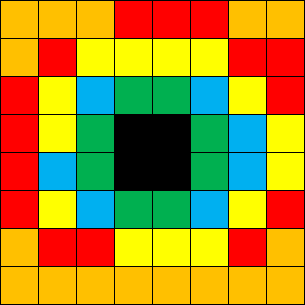

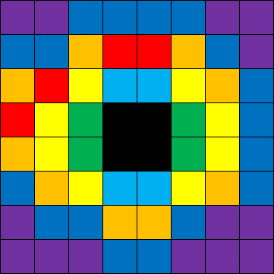

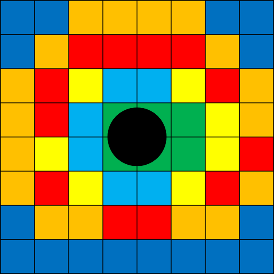

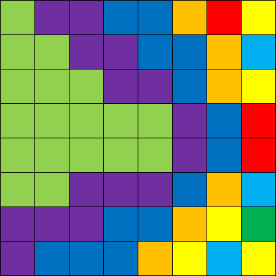


| **OPL** |  | **Singular group** | | | | | |
| --- | --- | --- | --- | --- | --- | --- | --- |
|  | C_1_ | 31.11 (0.3) | | | | | |
|  | C_2_ | 29.68 (0.39) | | | | | |
|  | C_3_ | 27.79 (0.26) | | | | | |
|  | C_4_ | 26.76 (0.35) | | | | | |
|  | C_5_ | 25.06 (0.49) | | | | | |
|  | C_6_ | 23.77 (0.29) | | | | | |
|  | C_7_ | 22.72 (0.28) | | | | | |
| **ONL_+HFL_** |  | **20-29 years** | **30-39 years** | **40-49 years** | **50-59 years** | **60-69 years** | **70+ years** |
|  | C_F_ | 84.55 (1.44) | 84.73 (1.85) | 84.59 (1.61) | 84.3 (1.95) | 84.57 (2.47) | 83 (1.51) |
|  | C_1_ | 70.14 (1.68) | 70.19 (1.75) | 69.27 (2.02) | 69.19 (1.5) | 68.87 (1.83) | 67.09 (1.7) |
|  | C_2_ | 61.4 (1.74) | 61.43 (2.1) | 60.49 (2.33) | 60.07 (2.59) | 60.06 (2.43) | 58.22 (2.41) |
|  | C_3_ | 54.56 (1.89) | 54.17 (1.93) | 53.11 (1.89) | 52.76 (2.05) | 52.86 (1.85) | 51.21 (1.75) |
|  | C_4_ | 48.02 (1.84) | 46.75 (1.88) | 46.47 (1.59) | 45.82 (1.59) | 45.88 (1.59) | 44.68 (1.76) |
| **IS/OS** |  | **20-29 years** | **30-39 years** | **40-49 years** | **50-59 years** | **60-69 years** | **70+ years** |
|  | C_F_ | 69.85 (0.23) | 69.13 (0.36) | 68.45 (0.57) | 68.71 (0.37) | 68.41 (0.26) | 66.68 (0.39) |
|  | C_1_ | 65.63 (0.65) | 64.74 (0.63) | 64.88 (0.47) | 64.59 (0.55) | 64.36 (0.51) | 63.82 (0.43) |
|  | C_2_ | 63.69 (0.53) | 63.1 (0.48) | 63.07 (0.58) | 63.07 (0.38) | 63.01 (0.43) | 63.02 (0.3) |
| **RPE** |  | **Singular group** | | | | | |
|  | C_F_ | 16.57 (0.45) | | | | | |
|  | C_1_ | 15.06 (0.11) | | | | | |
|  | C_2_ | 14.22 (0.16) | | | | | |
|  | C_3_ | 13.68 (0.1) | | | | | |
|  | C_4_ | 13.27 (0.11) | | | | | |
|  | C_5_ | 12.65 (0.17) | | | | | |
|  | **C_6_** | 12.03 (0.11) | | | | | |

**RPE**

**IS/OS**

**ONL_+HFL_**

**OPL**


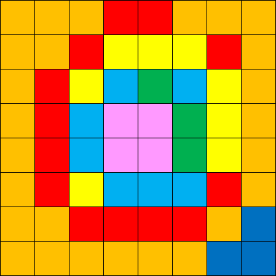

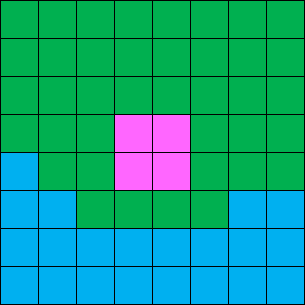

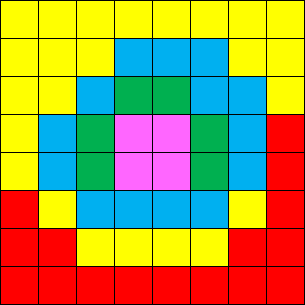

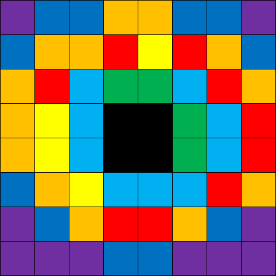

Supplement: Supplementary file 1 — Supplementary Tables. [file 41598_2020_79424_MOESM1_ESM.docx]
